# Regenerative growth is constrained by *brain tumor* to ensure proper patterning in *Drosophila*

**DOI:** 10.1371/journal.pgen.1011103

**Published:** 2023-12-21

**Authors:** Syeda Nayab Fatima Abidi, Felicity Ting-Yu Hsu, Rachel K. Smith-Bolton

**Affiliations:** 1 Department of Cell and Developmental Biology, University of Illinois at Urbana-Champaign, Urbana, Illinois, United States of America; 2 Carle R. Woese Institute for Genomic Biology, University of Illinois at Urbana-Champaign, Urbana, Illinois, United States of America; University of Colorado, UNITED STATES

## Abstract

Some animals respond to injury by inducing new growth to regenerate the lost structures. This regenerative growth must be carefully controlled and constrained to prevent aberrant growth and to allow correct organization of the regenerating tissue. However, the factors that restrict regenerative growth have not been identified. Using a genetic ablation system in the *Drosophila* wing imaginal disc, we have identified one mechanism that constrains regenerative growth, impairment of which also leads to erroneous patterning of the final appendage. Regenerating discs with reduced levels of the RNA-regulator Brain tumor (Brat) exhibit enhanced regeneration, but produce adult wings with disrupted margins that are missing extensive tracts of sensory bristles. In these mutants, aberrantly high expression of the pro-growth factor Myc and its downstream targets likely contributes to this loss of cell-fate specification. Thus, Brat constrains the expression of pro-regeneration genes and ensures that the regenerating tissue forms the proper final structure.

## Author summary

While much has been published about the signals that stimulate regeneration, the mechanisms that constrain and/or terminate regeneration have not been well characterized. Thus, we do not understand what limits may exist on the rate of regenerative growth, what mechanisms constrain regeneration, and what the consequences might be of enhancing regrowth. Here, we detail our discovery and characterization of a mechanism that constrains regeneration, and the deleterious effects of reducing that constraint. In this manuscript, we describe our identification of the RNA regulator Brat as a factor that constrains regenerative growth. Without this constraint on regenerative growth, patterning mistakes occur leading to a malformed regenerated structure. We demonstrate that the patterning errors are not due to faster growth itself, but may be due to the overexpression of the pro-growth transcription factor Myc.

## Introduction

Regeneration is the remarkable process by which some organisms replace tissues and organs after damage such that both morphology and function are restored. Complete regeneration requires several steps to occur correctly including wound healing, cell proliferation, and proper patterning and cell-fate specification in the newly formed tissue. The degree of regenerative capacity varies among different species, ranging from whole-body regeneration in hydra and planaria to limited tissue regeneration in mammals. Work in several model organisms has identified signaling pathways and molecular mechanisms that are important for initiating and executing regenerative growth after tissue damage, including JNK signaling [1–5], JAK/STAT signaling [6–8], EGFR signaling [9–12], Hippo signaling [13–17], Wnt signaling [18–24], and Myc [23,25]. Many of these mechanisms are also important during normal development, and the process of regeneration was traditionally thought to be a redeployment of earlier developmental steps [3,9,25–29]. However, recent evidence suggests that regeneration is not a simple reiteration of development but can employ regeneration-specific regulatory mechanisms [3,25,30–34]. Indeed, faithful regeneration likely requires additional mechanisms, since regrowth happens in the presence of wound-response signaling and in a developed juvenile or adult organism. Additionally, pro-growth pathways that are used during normal development are often activated in new ways and at higher strengths in the regenerating tissue [2,7,15,23]. These augmented pro-growth signals must be constrained as regeneration progresses to prevent aberrant growth and to enable re-establishment of pattern and cell-fate specification. Thus, growth suppressors and additional patterning factors are likely used to terminate regeneration and allow differentiation [35]. However, despite our understanding of the pro-growth signals needed for regeneration, we do not yet know what factors exist in different model organisms to restrain growth and promote re-patterning of regenerating tissue.

*Drosophila melanogaster* imaginal discs, precursors of adult fly appendages, are simple columnar epithelia that have well-characterized, complex expression of patterning genes that determine cell-fate specification. Imaginal discs undergo regeneration after damage [36], and we have previously used a genetic ablation system to study patterning in the regenerating tissue [23,32]. Here we identify the RNA-regulator Brain tumor (Brat) as a key constraint on regenerative growth that ensures proper formation of the regenerated structure. Brat is a member of the TRIM- (tripartite motif containing)-NHL (NCL-1, HT2A, and LIN-41) family of proteins and functions as a translational repressor by binding to its target RNAs either independently or in a complex with Pumilio and Nanos [37–39]. It acts as a potent differentiation factor and tumor suppressor in neural and ovarian germline stem cell lineages [40–43]. Human and mouse orthologs of Brat, TRIM3 and TRIM32 respectively, also possess tumor-suppressor activity in glioblastomas and are required for neuronal and muscle differentiation [44–47].

We show that regenerating wing imaginal discs with reduced levels of Brat regenerate better than controls, but the resulting adult wings have a disrupted margin. The margin loses some of the characteristic sensory bristles and veins, demonstrating an error in cell-fate specification. Importantly, these phenotypes are regeneration-specific, as they are not observed in the mutant animals after normal development. The enhanced regeneration appears due to increased expression of the growth regulators Myc and Wingless as well as upregulation of *ilp8*, which delays metamorphosis and allows the damaged tissue more time to regenerate. Intriguingly, Myc overexpression can also cause aberrant cell-fate specification at the wing margin. This disruption of patterning does not occur through general enhanced proliferation, but may be due to misregulation of Myc target genes, including *chronologically inappropriate morphogenesis* (*chinmo*). Hence, Brat constrains levels of Myc, Wg, and Ilp8, thereby acting as an important growth regulator and protective factor during regeneration, preventing errors in patterning, cell-fate specification, and differentiation in the regenerating tissue.

## Results

### Brat suppresses regenerative growth and is required for wing margin cell-fate specification during regeneration

**Fig 1:**
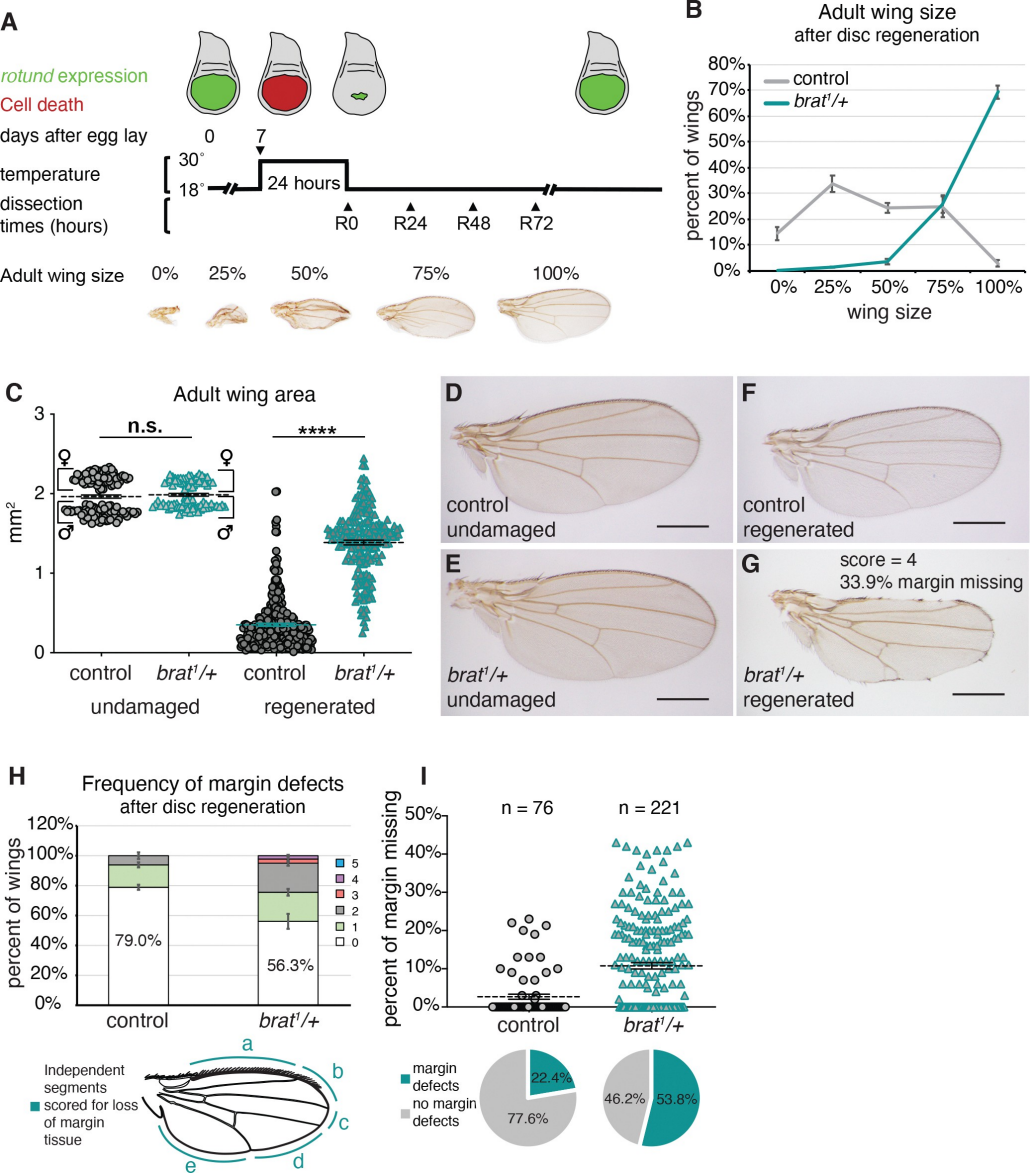
Enhanced regenerative growth and wing margin cell-fate specification defects in *brat*^*1*^*/+* during regeneration. (A) The protocol used to study regeneration. Animals were raised at 18°C and shifted to 30°C for 24 hours during early third-instar larval development on day 7 after egg lay (AEL). Larvae were returned to 18°C and were dissected at the time points noted during recovery (R) or allowed to pupariate and eclose. Representative wings depicting the range of adult wing sizes observed after regeneration compared to the size of a normal wing are shown. (B) Adult wing sizes observed after disc regeneration for control (*w*^*1118*^) (n = 317) and *brat*^*1*^*/+* (n = 208) wings, from three independent experiments. (C) Adult wing area after normal development or disc regeneration, measured using ImageJ after mounting and imaging wings, for control (*w*^*1118*^) (undamaged n = 133, regenerated n = 309) and *brat*^*1*^*/+* (undamaged n = 86, regenerated n = 195) wings. undamaged p = 0.45 regenerated p = 2.5158E-119. Wings in (C) are from the same experiments as (B). Note that number of wings in (C) is less for both control and *brat*^*1*^*/+* due to some wings being damaged during the mounting process. (D) Undamaged control (*w*^*1118*^) wing. (E) Undamaged *brat*^*1*^*/+* wing. (F) Adult control (*w*^*1118*^) wing after disc regeneration. (G) Adult *brat*^*1*^*/+* wing after disc regeneration. (H) Frequency of margin defects seen in adult wings after disc regeneration for control (*w*^*1118*^) (n = 93) and *brat*^*1*^*/+* (n = 218) wings, from three independent experiments. The wing margin was divided into five segments based on where the veins intersect the margin as shown in the diagram. Each wing was scored for the number of segments that had some margin tissue missing, with wings with a perfectly intact margin scoring at zero. Wing shown in (G) had tissue missing in four segments. (I) Margin tissue lost as a percentage of total wing perimeter for control (*w*^*1118*^) (n = 76) and *brat*^*1*^*/+* (n = 221) wings. p = 9.947E-08. The margin perimeter and the length of margin tissue lost were measured using ImageJ after mounting and imaging wings. Wings in (I) are from the same experiments as (H). Note that number of wings in the two quantifications is different because we did not quantify wings with length <1.1 mm for males and <1.7 mm for females, to ensure analysis was being carried out on nearly fully regenerated wings. (I). Percentage of wings with no defects fell from 79.0% to 77.6% for control and from 56.3% to 53.8% for *brat*^*1*^*/+* wings due to the increased ability to detect lost margin tissue at the higher magnification and resolution achieved by imaging the wings. Wing shown in (G) had 33.9% of margin tissue missing. Error bars mark standard error of the mean (SEM). Student’s T-test used for statistical analyses. Scale bars are 0.5 mm.

To identify genes important for regenerative growth and re-patterning, we performed a candidate screen, using our wing imaginal disc ablation system [23]. The primordial wing was targeted for ablation at the early third-instar larval stage by using *rotund-GAL4* to drive the expression of the proapoptotic gene *reaper* for 24 hours (Fig 1A). Our ability to restrict damage to 24 hours was provided by *tubulin-GAL80*^*ts*^, which can inhibit GAL4 activity at 18°C, but allows GAL4-driven cell death at 30°C in the 24-hour window. The extent of wing imaginal disc regeneration in the larvae was reflected in the adult wing size. Hence, the resulting adult wings were scored based on size and patterning features to identify mutants that affect genes that are involved in regulating regenerative growth and establishment of cell fates. There is inherent variability in this system because of environmental conditions such as temperature, humidity, and food quality that affect the pace of development, leading to minor differences in developmental age at the onset of ablation, causing the results of different experiments to vary slightly [14,48–51]. In addition, the average amount of regeneration across all animals can be modified by slightly shifting the timing of ablation, enabling us to choose experimental conditions that result in most control animals regenerating about half the wing primordium, to enable screening for enhanced or reduced regeneration in mutants. Animals with the same genotype within an experiment also showed some variation, due to stochastic differences in the time each animal takes to eclose, with animals that take longer to eclose having larger wings [23,50]. However, differences between control and mutant animals using this system are reproducible, consistent, and have identified key regeneration genes [32,49–51].

Using this genetic ablation system, we identified the gene *brain tumor (brat)* as an important regulator of regenerative growth. *brat*^*1*^*/+* [52,53] mutants that did not experience damage during development had adult wings that were not significantly different in size from controls across the whole population, although control female wings were slightly larger than *brat*^*1*^*/+* female wings (by an average of 0.03 mm^2^), and *brat*^*1*^*/+* male wings were slightly larger than control male wings (by an average of 0.09 mm^2^) (Figs 1C and S1A). However, after ablation and regeneration were induced, the combined population of *brat*^*1*^*/+* mutants showed enhanced regeneration and had adult wings that were, on average, much larger than controls that had also undergone regeneration (an average of difference of 1.04 mm^2^) (Fig 1B and 1C). We confirmed this enhanced regeneration phenotype in heterozygotes for three other *brat* mutant alleles: *brat*^*192*^, *brat*^*150*^ [54] and *brat*^*11*^ [55](S1B Fig). Each of these ethylmethonylsulfate-induced mutations is considered amorphic, and the molecular lesions in *brat*^*11*^, *brat*^*192*^, and *brat*^*150*^ are premature stop codons [40, 42].

Interestingly, we also discovered a role for *brat* in cell-fate specification during regeneration. After normal development, *brat*^*1*^*/+* mutants had adult wings that were patterned normally (Figs 1D, 1E and S1C). To confirm that reduction in *brat* expression does not cause patterning errors during normal development, we knocked down Brat levels in the entire wing pouch using *brat* RNAi, which resulted in adult wings that were patterned normally (S1D and S1E Fig). A previous study in which Brat levels were reduced in the anterior and posterior compartments of the wing also did not report any patterning defects [56]. However, when discs were ablated and allowed to regenerate, *brat* heterozygous mutant wings that had regenerated to 75% or 100% size showed aberrant patterning such that the wing margin lost sensory bristles and vein material (Fig 1F and 1G). By contrast, control regenerated wings that had regenerated to 75% or 100% size lost margin tissue at a lower frequency (Fig 1H and 1I). Furthermore, the extent of margin tissue lost was not as severe in control regenerated wings as compared to *brat*^*1*^*/+* regenerated wings (Fig 1H and 1I). Similar to the enhanced regeneration seen in *brat* mutants, we confirmed the loss-of-margin defect in heterozygotes for the additional three mutant alleles (S1F Fig). We attempted to reduce Brat levels solely in the regenerating wing pouch, and found that Brat RNAi failed to reduce Brat expression during regeneration (S1G Fig) despite its ability to reduce Brat levels during normal development (S1D Fig). Therefore, we were unable to rule out a non-autonomous requirement for Brat outside of the regenerating tissue.

### *brat* regulates entry into metamorphosis

**Fig 2:**
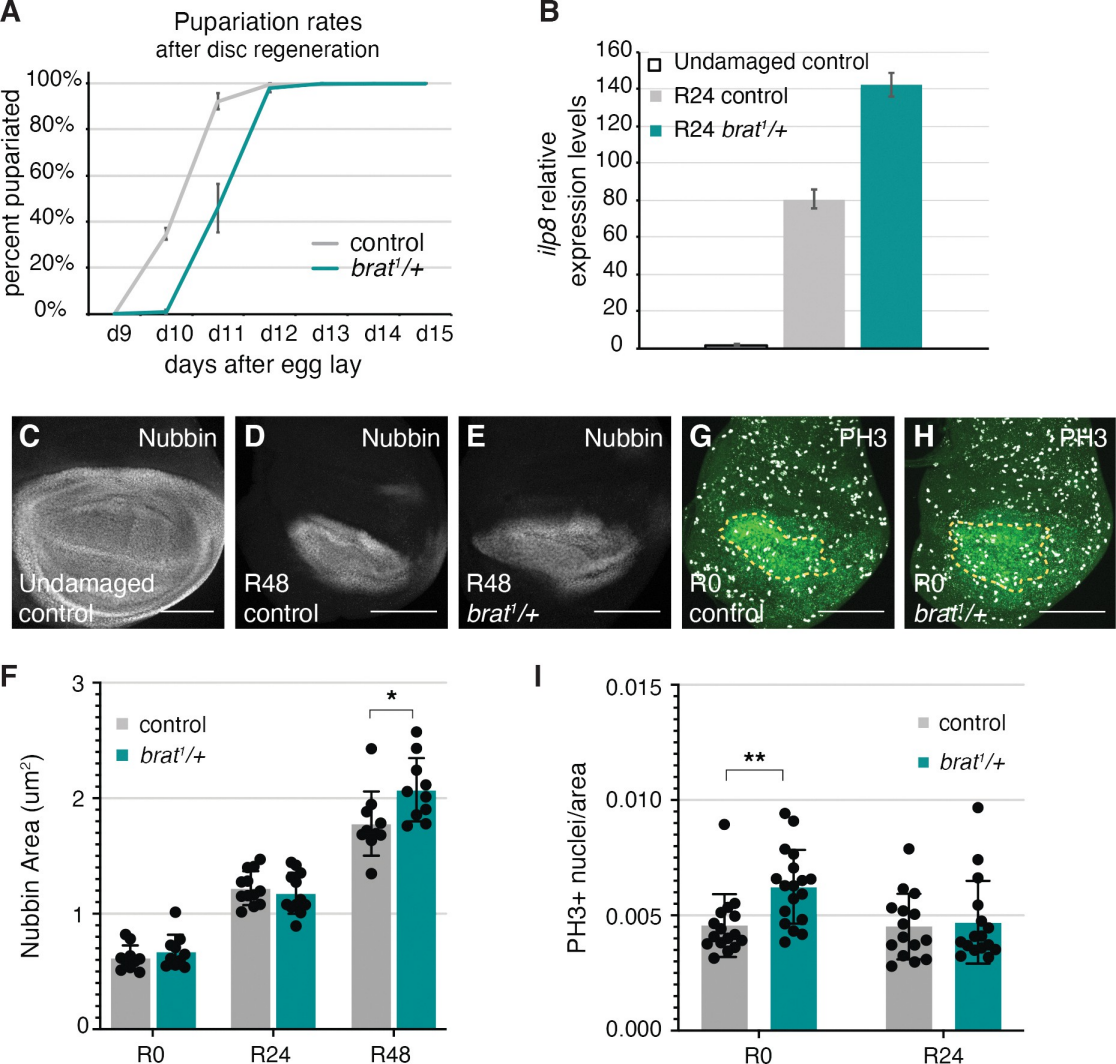
*brat*^*1*^*/+* animals have a regenerative growth advantage. (A) Pupariation rates after disc regeneration for control (*w*^*1118*^) (n = 384) and *brat*^*1*^*/+* (n = 107) animals, from three independent experiments. (B) Relative expression levels of *ilp8* for undamaged control, R24 control (*w*^*1118*^) and R24 *brat*^*1*^*/+* discs. (C) Anti-Nubbin immunostaining in an undamaged control disc. (D-E) Anti-Nubbin immunostaining in an R48 control (*w*^*1118*^) disc (D), and an R48 *brat*^*1*^*/+* disc (E). (F) Quantification of area of Nubbin-expressing cells for control (*w*^*1118*^) and *brat*^*1*^*/+* discs at R0 (n = 10 and 10), R24 (n = 12 and 12) and R48 (n = 10 and 10). * p < 0.03. (G-H) Anti-PH3 immunostaining (white) and anti-Nubbin (green) in an R0 control (*w*^*1118*^) disc (G), and an R0 *brat*^*1*^*/+* disc (H). The yellow dashed lines outline the Nubbin-expressing wing pouch. (I) PH3-positive nuclei were counted within the regenerating tissue as marked by Anti-Nubbin co-immunostaining. Quantification of PH3-positive nuclei in Nubbin area for control (*w*^*1118*^) and *brat*^*1*^*/+* discs at R0 (n = 16 and 18) and R24 (n = 15 and 16). ** p < 0.002. Error bars represent SEM. Student’s T-test used for statistical analyses. Scale bars are 100 μm.

Tissue damage in imaginal discs can induce a systemic response in the larvae, which extends the larval phase of development and delays pupariation [23,57]. This delay in pupariation is due to expression of the relaxin-like peptide *ilp8* in damaged discs [58,59]. To determine whether *brat* mutants regenerated better due to an enhanced delay in pupariation, we measured rates of pupariation in control and mutant animals. We found that during normal development, control and *brat*^*1*^*/+* animals pupariated at the same time, indicating that the two genotypes develop at similar rates (S2A Fig). After disc damage, *brat* mutants delayed pupariation an additional day compared to controls in which discs were also damaged (Figs 2A and S2B). Note that direct comparisons cannot be made between regenerating larvae that spent 24 hours at 30°C (Figs 2A and S2B) and normally developing larvae that remain at 18°C (S2A Fig), due to the effects of temperature on development. Our data show that *brat/+* mutants are able to stay in the larval stage even longer than controls, giving them more time to regenerate. Interestingly, when we measured *ilp8* transcript levels through qPCR, we saw an 80-fold increase in *ilp8* levels after tissue damage in controls, and a 140-fold increase in *brat*^*1*^*/+* animals (Fig 2B), suggesting that Brat’s effects on pupariation timing could be through Ilp8 expression.

### *brat* restricts growth and proliferation during regeneration

Regenerative growth occurs through localized cell proliferation at the wound site [23,60].The proliferating cells, known as the blastema, give rise to the regenerated tissue. The blastema and the subsequent regenerated wing pouch can be labeled with the wing primordium marker Nubbin (Nub) [61]. To determine whether *brat*^*1*^*/+* discs regenerated better due to increased growth rates in the wing pouch, we measured the area of the Nub-expressing cells in control and *brat*^*1*^*/+* regenerating discs. In the initial stages of regeneration, the control and mutant had similar Nub-expressing areas and numbers of Nub-expressing cells, indicating equal ablation and equal early regrowth. However, by 48 hours after tissue damage (recovery time 48, or R48), *brat*^*1*^*/+* wing discs had a significantly bigger Nub-expressing pouch than the control (Figs 2D–2F, S2C), indicating that *brat/+* mutants were regenerating faster than controls. To assess whether this difference in growth rates was due to differences in proliferation, we counted cells going through mitosis by quantifying Phospho-histone H3 (PH3)-positive nuclei in the regenerating blastema. Reduction of *brat* resulted in a significantly higher number of PH3-positive nuclei per area at R0, but this increased proliferation had subsided to normal levels by R24 (Fig 2G–2I). To confirm that the increased number of PH3-positive nuclei were not simply due to an increased number of total cells in the blastema, we also counted total Nubbin-positive nuclei in the regenerating blastema to calculate a ratio of proliferating cells to blastema cells (S2D Fig). In addition, EdU incorporation was increased in the *brat*^*1*^*/+* regenerating wing pouch relative to the notum at R0 (S2E Fig). These data confirm increased proliferation in *brat*^*1*^*/+* regenerating discs at R0. We have observed previously that it can take time for a slight difference in proliferation in the regenerating tissue to result in a measurable difference in the wing pouch area. For example, animals heterozygous for *cap-n-collar* had reduced numbers of PH3-immunostaining nuclei at R0 but did not show a statistically significant difference in pouch size until R48 [51]. Therefore, reduction of *brat* gives the regenerating tissue a growth advantage early in regeneration, resulting in a measurable difference in tissue area by R48.

**Fig 3:**
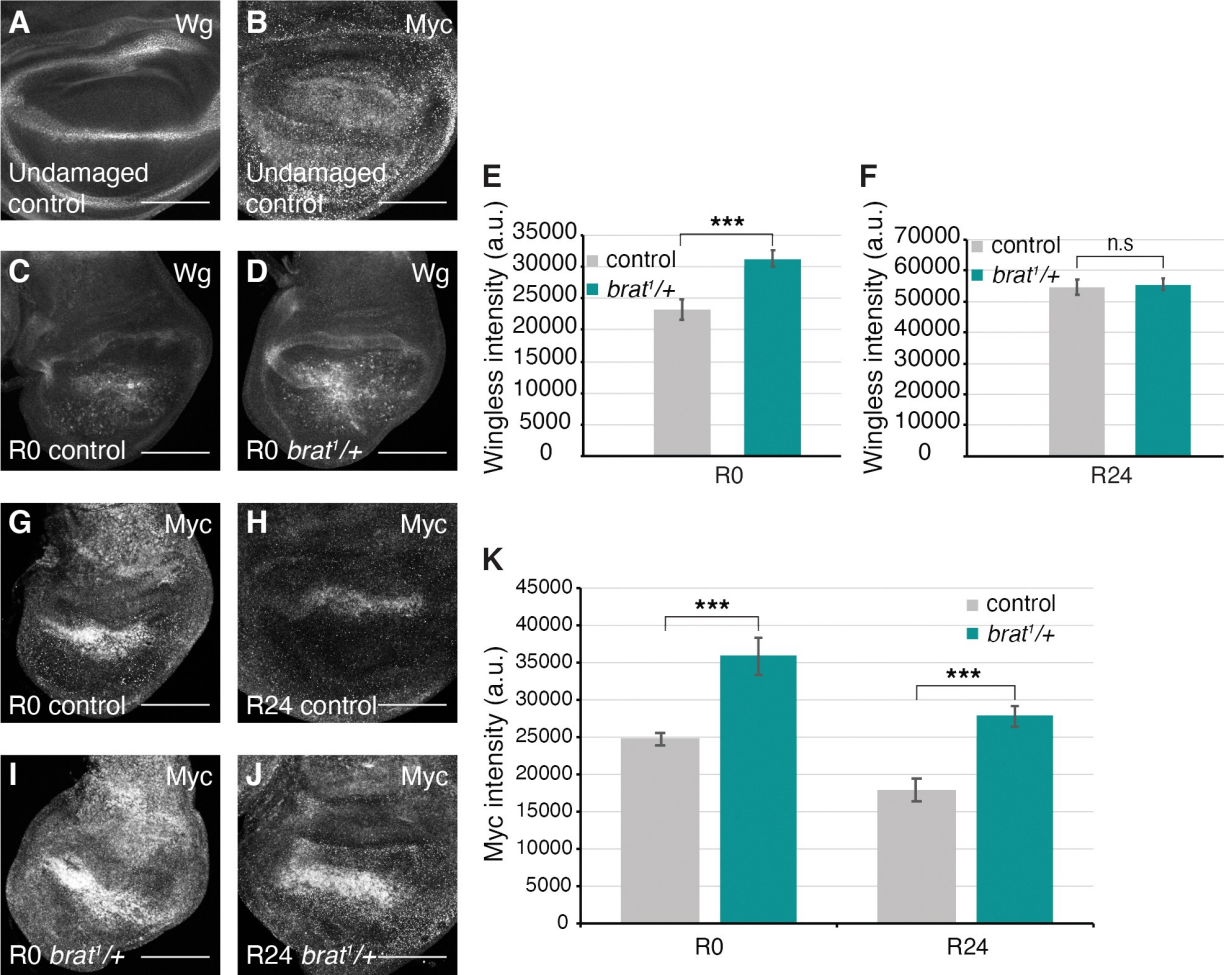
*brat*^*1*^*/+* animals experience elevated regeneration signaling. (A) Anti-Wg immunostaining in an undamaged control (*w*^*1118*^) disc. (B) Anti-Myc immunostaining in an undamaged control (*w*^*1118*^) disc. (C-D) Anti-Wg immunostaining in an R0 control (*w*^*1118*^) disc (C) and an R0 *brat*^*1*^*/+* disc (D). (E) Quantification of Wg fluorescence intensity in R0 control (*w*^*1118*^) (n = 13) and R0 *brat*^*1*^*/+* (n = 17) discs. *** p < 0.0006. (F) Quantification of Wg fluorescence intensity in R24 control (*w*^*1118*^) (n = 12) and R24 *brat*^*1*^*/+* (n = 11) discs. Area for fluorescence intensity measurement was defined by the Wg expression domain in the wing pouch. (G-J) Anti-Myc immunostaining in an R0 control (*w*^*1118*^) disc (G), an R24 control (*w*^*1118*^) disc (H), an R0 *brat*^*1*^*/+* disc (I) and an R24 *brat*^*1*^*/+* disc (J). (K) Quantification of Myc fluorescence intensity in R0 control (*w*^*1118*^) (n = 13), R0 *brat*^*1*^*/+* (n = 12), R24 control (*w*^*1118*^) (n = 13), and R24 *brat*^*1*^*/+* (n = 12) discs. Area for fluorescence intensity measurement was defined by the elevated Myc expression domain in the wing pouch. R0 *** p < 0.0003, R24 *** p < 0.0001. Error bars represent SEM. Student’s T-test used for statistical analyses. Scale bars are 100 μm.

Wingless (Wg) and Myc are regulators of regenerative growth and are upregulated at the wound site after damage [20,21,23]. Interestingly, Brat regulates stem cell differentiation in the brain by suppressing self-renewal signaling pathways such as Wnt signaling, and acting as a post-transcriptional inhibitor of Myc, to enable specification of progenitor cell fate [42,62]. Additionally, Brat overexpression can suppress Myc at the post-transcriptional level in wing disc epithelial cells, although loss of *brat* does not lead to elevated Myc protein in wing discs during normal development [56]. To determine whether these regulators of regenerative growth are upregulated in *brat*^*1*^*/+* regenerating discs, we examined the expression of Wg and Myc. Wg is normally expressed along the Dorso-ventral (DV) boundary and in two concentric circles at the inner and outer edge of the wing pouch [63] (Fig 3A), and Myc is expressed in the wing pouch, but is repressed in the cells at the DV boundary as they undergo cell cycle and growth arrest [64] (Fig 3B). Both Wg and Myc expression were comparable to controls in undamaged *brat*^*1*^*/+* discs (S3A–S3E Fig). When damage is induced, Wg is upregulated throughout the blastema by R0 [23] (Fig 3C). Reduction of *brat* expression resulted in significantly higher levels of Wg expression at R0 (Fig 3D and 3E) but not at R24 (Fig 3F). After ablation, Myc expression is elevated in the regenerating tissue [23] (Fig 3G and 3H). *brat*^*1*^*/+* discs showed significantly higher levels of Myc at R0, which were sustained through R24 (Fig 3I–3K). Thus, being heterozygous mutant for *brat* caused an increase in the levels of both Wg and Myc early in regeneration. The elevated expression of these growth regulators likely explains the high proliferation seen in *brat*^*1*^*/+* discs at R0, and the larger wing pouch at R48.

### *brat* is required for margin cell-fate specification during regeneration

**Fig 4:**
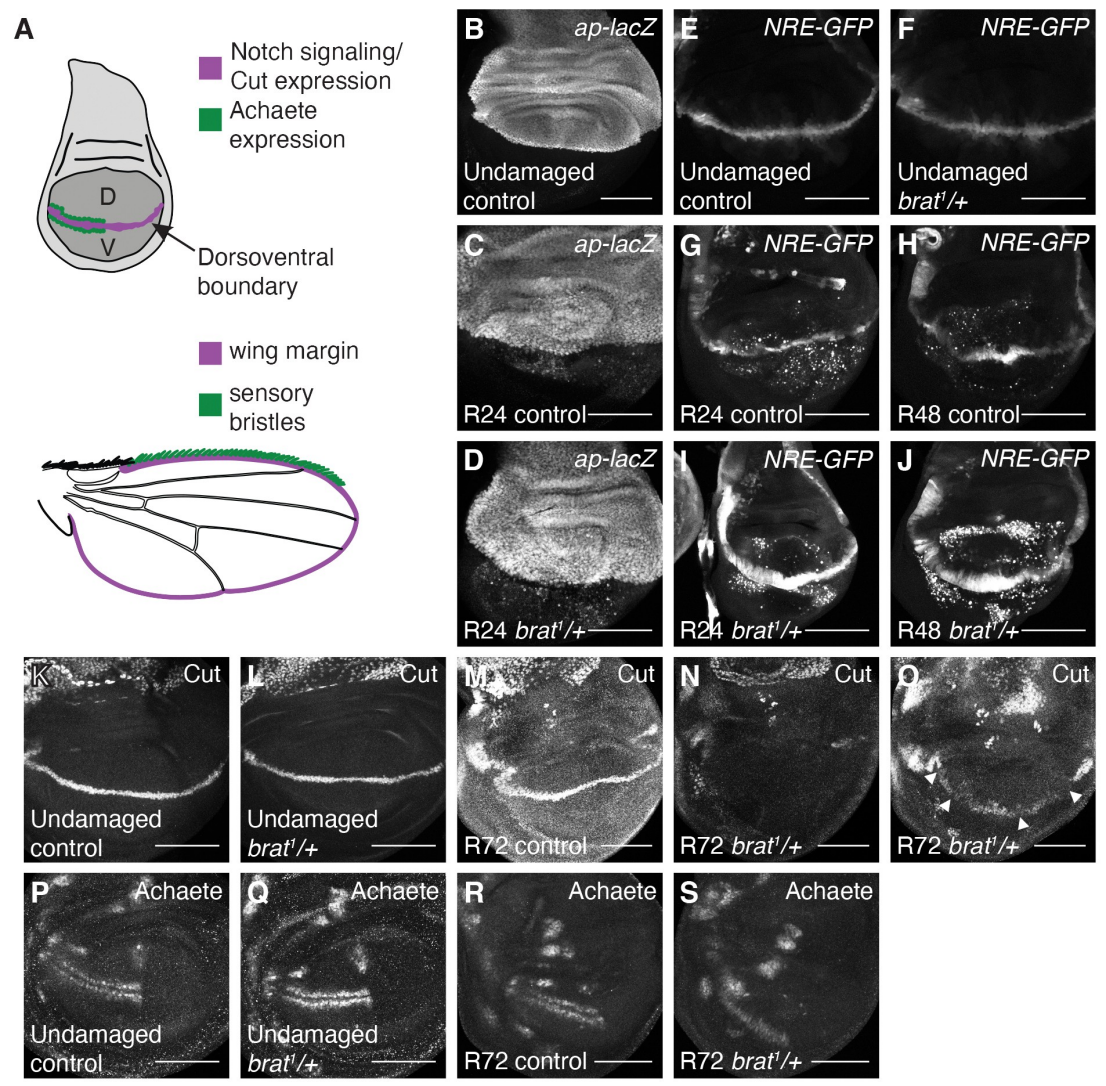
*Brat* regulates margin cell-fate specification. (A) Drawings of a wing imaginal disc and an adult wing. D = dorsal and V = ventral compartments of the wing disc, with the dorsoventral boundary marked in purple. Notch signaling and Cut expression are present at the dorsoventral boundary, which forms the adult wing margin, also marked in purple. Achaete-expressing cells, marked in green, give rise to the sensory bristles at the anterior half of the margin in the adult wing, also marked in green. (B) *ap-lacZ* expression in an undamaged control disc from a third-instar *ap-lacZ/CyO* animal. (C-D) *ap-lacZ* expression in an R24 control (*w*^*1118*^) disc (C) and an R24 *brat*^*1*^*/+* disc (D). (E-F) *NRE-GFP* expression in an undamaged control (*w*^*1118*^) disc (E) and an undamaged *brat*^*1*^*/+* disc (F). (G-J) *NRE-GFP* expression in an R0 control (*w*^*1118*^) disc (G), an R24 control (*w*^*1118*^) disc (H), an R0 *brat*^*1*^*/+* disc (I) and an R24 *brat*^*1*^*/+* disc (J). (K-L) Anti-Ct immunostaining in an undamaged control (*w*^*1118*^) disc (K) and an undamaged *brat*^*1*^*/+* disc (L). (M-O) Anti-Ct immunostaining in an R72 control (*w*^*1118*^) disc (M) and an R72 *brat*^*1*^*/+* discs (N-O). Arrowheads point to loss of Ct expression in (O). (P-Q) Anti-Ac immunostaining in an undamaged control (*w*^*1118*^) disc (P) and an undamaged *brat*^*1*^*/+* disc (Q). (R-S) Anti-Ac immunostaining in an R72 control (*w*^*1118*^) disc (R) and an R72 *brat*^*1*^*/+* disc (S). Scale bars are 100 μm.

Reduction of *brat* during regeneration caused patterning defects specifically at the wing margin, resulting in the loss of vein at the margin and loss of sensory bristles (Fig 1G). Thus, *brat* is required for correct cell-fate specification at the DV boundary during regeneration. The wing imaginal disc is divided into the dorsal and the ventral compartments, with expression of the LIM-homeodomain protein Apterous (Ap) in dorsal cells. The juxtaposition of the dorsal and ventral cells forms the DV boundary, which develops into the adult wing margin [65] (Fig 4A). Notch (N) and Wg signaling at the DV boundary are crucial for the correct organization and cell-fate specification at the boundary [66]. *cut (ct)* and *achaete (ac)* are margin-specific genes that are expressed downstream of N and Wg signaling. *ct* is required for the specification of the wing margin, and *ac* specifies the pro-neural sensory organ precursors [66,67] (Fig 4A).

To investigate whether the errors in fate specification seen in *brat*^*1*^*/+* discs were due to a compromised compartment boundary, we examined the expression of Ap using the *ap-lacZ* reporter. *ap-lacZ* expression showed a clear DV boundary in the undamaged control discs (Fig 4B). The DV boundary remained intact after ablation in control and *brat*^*1*^*/+* discs (Figs 4C, 4D, S4A and S4B). *ap-lacZ* expression was also seen in the debris found in the damaged wing imaginal disc, due to the perdurance of β-gal. Wg expression was restored to its normal DV expression by R48 in both control and *brat*^*1*^*/+* discs (S4C and S4D Fig), and overexpression of Wg did not cause margin defects (S3F Fig). Therefore, the patterning defects were not caused by disruptions in the DV boundary or changes in Wg expression.

Next, we examined N signaling in *brat*^*1*^*/+* discs due to its critical role in specifying fates at the DV boundary. We used a N signaling reporter, which uses *Notch Response Elements* (*NREs*) that bind to the Notch co-receptor Suppressor of Hairless, to drive the expression of GFP [68]. No difference was detected in the expression of the N reporter in undamaged control and *brat*^*1*^*/+* discs (Fig 4E and 4F). N signaling at the DV boundary was restored by R24 in controls and continued at R48 (Fig 4G and 4H). Note that the reporter signal can also be seen in cellular debris in the regenerating discs due to the perdurance of GFP. Interestingly, *brat*^*1*^*/+* discs showed elevated levels of the N signaling reporter at both these time points (Fig 4I and 4J). This result is consistent with recent evidence demonstrating Brat’s ability to attenuate N nuclear transport in the brain [69]. We wondered whether this elevated N signaling could also disrupt margin fates. However, overexpressing the N-intracellular domain in the wing pouch during the 24-hour ablation period (S4E and S4F Fig) resulted in adult wings that were patterned remarkably well, with significantly fewer wings showing any margin defects when compared to the control (S4G Fig). Thus, increased N activity during regeneration suppresses margin defects. Additionally, decreasing N signaling activity in *brat*^*1*^*/+* regenerating discs by using a mutation in the *anterior pharynx defective 1 (aph-1)* gene was unable to rescue the *brat* heterozygous phenotype. *aph-1*^*D35*^*/+* discs showed significantly reduced N signaling during normal development (S4H–S4J Fig) and at R24 during regeneration (S4K–S4M Fig), but could not rescue the loss of margin phenotype in the *brat* mutant (S4N Fig). Thus, while Brat constrains N signaling during regeneration, the elevated N signaling in *brat*^*1*^*/+* mutants does not cause the margin cell-fate specification defects.

To determine whether the loss of margin cell fates was due to apoptosis of those cells later in regeneration, we immunostained regenerating discs for the cleaved caspase Dcp-1 as a marker of cell death, and Wg to mark the margin. We did not observe dying cells in the margin in either control or *brat*^*1*^*/+* regenerating discs (S4C and S4D Fig).

### *brat* specifies margin fate by controlling the expression of Cut and Achaete

To understand how patterning was disrupted in *brat*^*1*^*/+* regenerating discs, we examined expression of margin cell-fate genes. Cut (Ct) expression was present along the DV boundary in both undamaged control and *brat*^*1*^*/+* discs (Fig 4K and 4L), consistent with our results showing that adult undamaged *brat*^*1*^*/+* wings do not have margin defects (S1D Fig). In control regenerating discs, Ct expression was detected at the DV boundary at R72, which is when regeneration and repatterning are largely complete (Fig 4M). By contrast, Ct expression was either not observed in *brat*^*1*^*/+* discs or was still missing in segments of the DV boundary at R72 (Fig 4N and 4O). These results indicate a specific error in cell-fate specification, as the DV boundary was intact at R72 (S4A and S4B Fig). Undamaged control and *brat*^*1*^*/+* discs also showed appropriate Ac expression in two stripes of cells directly flanking the DV boundary in the anterior half of the disc (Fig 4P and 4Q). Ac expression was also detected in control regenerating discs at R72 (Fig 4R). While Ac-expressing cells appeared in *brat*^*1*^*/+* discs, they were not clearly separated across the DV boundary (S4 Fig). This finding is consistent with previous reports showing that Ct suppresses Ac at the margin, and mutations in *ct* lead to aberrant expression of Ac at the DV boundary, followed by degeneration of the wing margin and loss of the developing sensory bristles between 24 and 36 hours after puparium formation [70,71].

### High Myc expression perturbs margin cell-fate specification during regeneration

Our results show that Brat both restricts regenerative growth and ensures correct cell-fate specification at the wing margin, but it was unclear whether these phenomena were linked. Interestingly, we have previously shown that a pro-regeneration signal can have deleterious effects on cell fate, and that the regenerating tissue deploys a protective factor to minimize regeneration-induced cell-fate disruption. JNK signaling in regenerating tissue can cause aberrant posterior-to-anterior cell-fate changes, which can be suppressed by a regeneration-specific protective factor, Taranis, to ensure correct patterning of the regenerating tissue [32]. Therefore, we wondered whether unconstrained regenerative growth, or unconstrained expression of growth drivers, could also have deleterious side effects such as loss of margin cell fates. We have ruled out elevated N signaling as the causative factor for the cell-fate errors that occurred in *brat*^*1*^*/+* regenerating discs (S4 Fig). Furthermore, overexpression of *wg* or *ilp8* did not cause margin defects (S3F and S3G Fig). Therefore, we wondered whether high Myc levels caused by reduced Brat could cause the margin defects.

**Fig 5:**
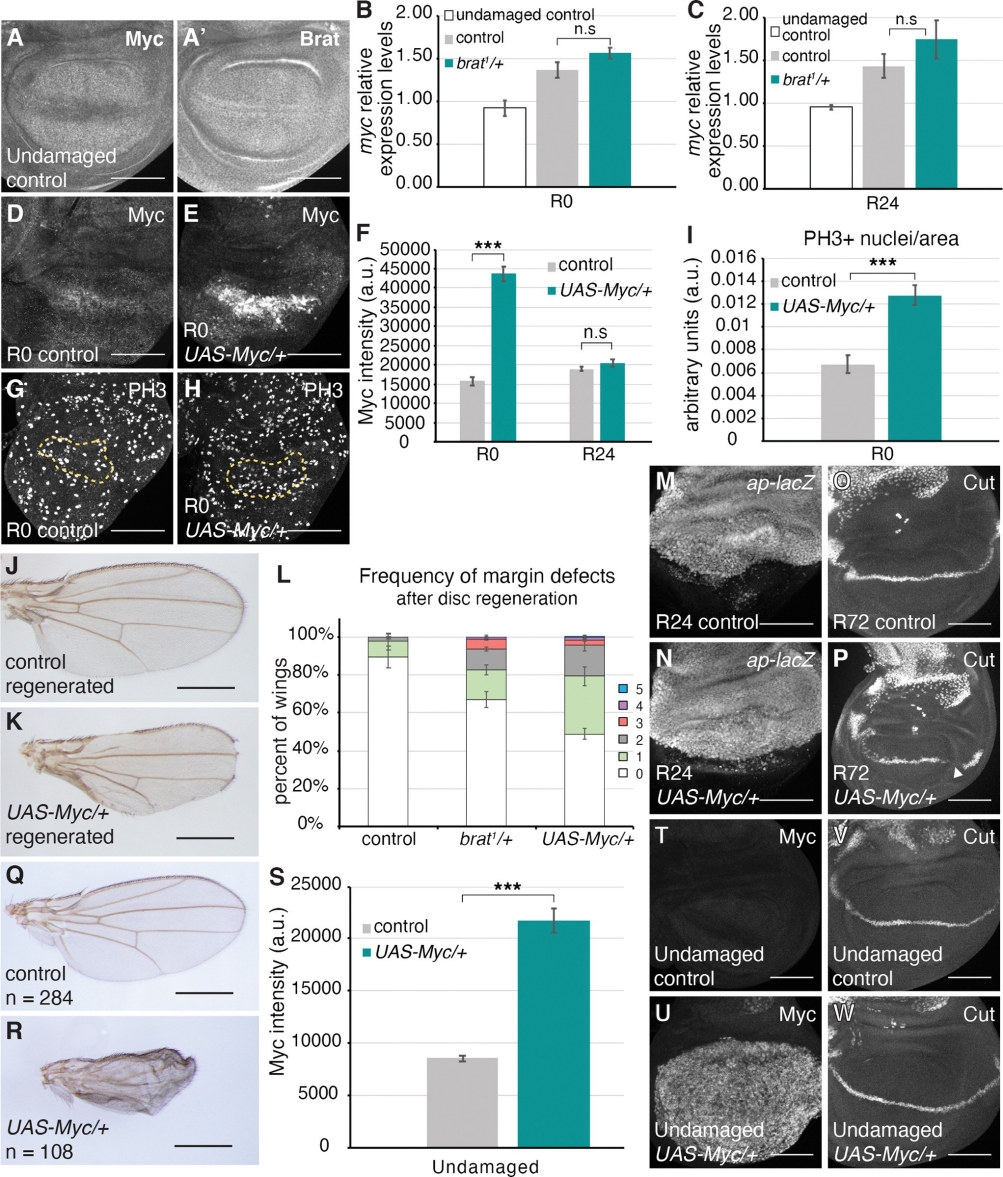
High Myc expression causes margin defects. (A-A’) Anti-Myc and Anti-Brat co-immunostaining in an undamaged control disc. *rnGAL4*, *GAL80*^*ts*^*/attP2* animals were shifted to 30°C on day 7 AEL and dissected 24 hours later. (B-C) Relative expression levels of *myc* for undamaged control, regenerating control (*w*^*1118*^), and regenerating *brat*^*1*^*/+* discs at R0 (B) and R24 (C). P values for comparison between regenerating control and *brat*^*1*^*/+* discs: p > 0.1 at R0, and p > 0.3 at R24. (D-E) Anti-Myc immunostaining in an R0 control (*w*^*1118*^) disc (D) and an R0 *UAS-Myc/+* disc (E). (F) Quantification of Myc fluorescence intensity in R0 control (*w*^*1118*^) (n = 13), R0 *UAS-Myc/+* (n = 12), R24 control (*w*^*1118*^) (n = 13), and R24 *UAS-Myc/+* (n = 12) discs. Area for fluorescence intensity measurement was defined by the elevated Myc expression domain in the wing pouch. *** p = 1.2E-11. (G-H) Anti-PH3 immunostaining in an R0 control (*w*^*1118*^) disc (G), and an R0 *UAS-Myc/+* disc (H). The yellow dashed lines outline the Nubbin-expressing wing pouch. (I) PH3-positive nuclei were counted within the regenerating wing pouch as marked by Anti-Nubbin co-immunostaining. Quantification of PH3-positive nuclei in the Nubbin area for R0 control (*w*^*1118*^) (n = 15) and *UAS-Myc/+* (n = 15) discs. *** p < 0.00002. (J) Adult control (*w*^*1118*^) wing after disc regeneration. (K) Adult *UAS-Myc/+* wing after disc regeneration. (L) Frequency of margin defects, as quantified in Fig 1H, seen in adult wings after disc regeneration for control (*w*^*1118*^) (n = 134), *brat*^*1*^*/+* (n = 193) and *UAS-Myc/+* (n = 200) wings, from three independent experiments. (M-N) *ap-lacZ* expression in an R24 control (*w*^*1118*^) disc (M) and an R24 *UAS-Myc/+* disc (N). (O-P) Anti-Ct immunostaining in an R72 control (*w*^*1118*^) disc (O) and an R72 *UAS-Myc/+* disc (P). (Q) Adult undamaged control (*+; rnGAL4*,*GAL80ts/+*) wing from animals shifted to 30°C on day 7 AEL and maintained at 30°C until eclosion. (R) Adult wing from discs continuously overexpressing Myc from day 7 AEL onwards (*UAS-Myc/+; rnGAL4*, *GAL80ts/+)*. (S) Quantification of Myc fluorescence intensity in undamaged discs dissected 31 hours after animals were shifted to 30°C on day 7 AEL. Control (*+; rnGAL4*,*GAL80ts/+*) (n = 14) and *UAS-Myc (UAS-Myc/+; rnGAL4*, *GAL80ts/+)* (n = 14). Area for fluorescence intensity measurement was defined by the Myc expression domain in the wing pouch. *** p = 1.2E-11. (T-U) Anti-Myc immunostaining in an undamaged control disc (T) and an undamaged *UAS-Myc/+* disc (U). (V-W) Anti-Ct immunostaining in an undamaged control disc (V) and an undamaged *UAS-Myc/+* disc (W). Error bars represent SEM. Student’s T-test used for statistical analyses. Scale bars are 100 μm. Scale bars for adult wings are 0.5 mm.

Brat overexpression can suppress Myc in wing imaginal disc cells [56], and in undamaged wing discs Brat protein levels were elevated at the DV boundary where Myc was reduced (Fig 5A and 5A’), suggesting that Brat may regulate Myc at the DV boundary. Furthermore, our results showed that regenerating *brat/+* mutant discs experienced elevated Myc levels compared to controls (Fig 3G–3K). Previous studies demonstrated that Brat regulates Myc at the post-transcriptional level [56]. To confirm that Brat is also regulating *myc* post-transcriptionally during regeneration, we measured *myc* transcript levels through qPCR. Regenerating discs showed significantly increased transcription of *myc* compared to undamaged controls. However, there was no significant difference in *myc* transcript levels between regenerating control and *brat*^*1*^*/+* discs at R0 and R24 (Fig 5B and 5C), indicating that Brat’s regulation of Myc must be post-transcriptional.

To test whether the elevated Myc protein levels could cause margin defects during regeneration and phenocopy the *brat* mutation, we overexpressed Myc in the wing pouch during the 24-hour ablation period. Myc was highly upregulated at R0 (Fig 5D–5F), but Myc levels had returned to normal by R24 (Fig 5F). Overexpression of Myc also resulted in a significantly higher number of proliferating nuclei in the regenerating tissue at R0, similar to *brat*^*1*^*/+* discs (Fig 5G–5I). Remarkably, we observed that adult wings resulting from Myc-overexpressing regenerating discs also showed margin defects similar to the *brat*^*1*^*/+* wings (Fig 5J and 5K). Moreover, the frequency of margin defects in the adult wings resulting from Myc-overexpressing regenerating discs was even higher than in adult wings resulting from *brat*^*1*^*/+* regenerating discs (Fig 5L). This difference in severity of phenotype correlates with levels of Myc at R0; in *brat*^*1*^*/+* regenerating discs Myc levels are higher than the control by 1.4 and 1.5-fold at R0 and R24 respectively (Fig 3K), and in Myc-overexpressing regenerating discs, Myc levels are higher than control by 2.75 fold at R0 but become similar to controls by R24 (Fig 5F). Thus, elevated levels of Myc alone can cause errors in margin cell-fate specification during regeneration. Similar to *brat*^*1*^*/+* discs, *ap-lacZ* expression showed that the compartment boundary was not compromised in Myc-overexpressing regenerating discs (Fig 5M and 5N). However, Ct expression was missing in segments at the DV boundary as in the *brat*^*1*^*/+* discs (Fig 5O and 5P).

Overexpressing Myc for a 24-hour window during normal development resulted in 3 adult wings out of 730 that showed any margin defects (S5A Fig). Even in these wings, only one segment of the margin was affected. To rule out the possibility that transient overexpression of Myc during development may not be sufficient to perturb patterning, we overexpressed Myc continuously after the animals entered the third instar larval stage. Continuous Myc overexpression proved to be lethal for many animals. While the flies that survived had significantly smaller wings, the margins showed no defect (Fig 5Q and 5R). 31 hours of Myc overexpression during normal development showed significantly higher Myc protein levels (Fig 5S–5U) but did not interfere with Ct expression (Fig 5V and 5W). These data indicate that high Myc levels do not cause cell-fate specification errors during normal development, and the extensive loss of wing margin induced by high Myc expression is a regeneration-specific phenotype.

### Loss of Myc expression also perturbs margin cell-fate specification during regeneration

We hypothesized that if the *brat* phenotype was due to elevated Myc levels, we would be able to rescue the phenotype by reducing Myc levels in the *brat* mutant. For this purpose, we used *dm*^*4*^, which is a null allele of Myc [72]. Surprisingly, we observed that the *dm*^*4*^*/+* mutants alone showed margin defects in the regenerated wings at a frequency similar to *brat*^*1*^*/+*, even though the *dm*^*4*^*/+; brat*^*1*^*/+* double mutant showed slightly reduced frequency of margin defects (S5B Fig). To confirm that Myc levels were reduced in the *dm*^*4*^*/+* mutants, we quantified Myc protein through immunostaining. We observed that there was no significant difference in Myc expression levels between the *dm*^*4*^*/+* mutant and control, both during development and regeneration (S5C and S5D Fig). Indeed, Myc levels were trending higher in the *dm*^*4*^*/+* discs during regeneration, perhaps accounting for the margin defects. The failure of the *dm*^*4*^ mutation to reduce Myc levels could be due to compensatory expression of the functional copy of the Myc locus. We next tried reducing Myc levels though RNAi. Despite the RNAi expression being transient in our system, and only occurring in cells that survive ablation, RNAi-mediated persistent knockdown has worked for multiple genes, likely due to the shadow RNAi effect [73]. Two RNAi lines could significantly reduce Myc levels during normal development when expressed during early third instar (S5E Fig). However, when Myc RNAi was expressed during the 24-hour ablation period, Myc levels were not reduced at either R0 or R24, with one Myc RNAi line showing significantly higher levels of Myc compared to the control (S5F Fig).

To reduce Myc levels without the possibility of compensatory expression from an endogenous allele, we turned to the viable hypomorphic allele *Myc*^*P0*^, which is reported to eliminate Myc mRNA in the wing pouch [74]. Indeed, we found that male larvae hemizygous for *Myc*^*P0*^ did not show the typical Myc expression in third-instar wing discs, and lacked upregulation of Myc during regeneration (S5G and S5H Fig). The *Myc*^*P0*^ males developed more slowly than control larvae, so we adjusted the timing of induction of ablation later by 4 hours to coincide with the early third instar larval phase. Surprisingly, we found that *Myc*^*P0*^ males that had undergone ablation and regeneration in the wing discs also had adult wings that were missing substantial sections of their margins, as did *Myc*^*P0*^; *brat*^*1*^*/+* double mutants (S5I Fig). Indeed, immunostaining *Myc*^*P0*^ regenerating discs showed gaps in *cut* expression, indicating disruption of margin fate (S5J and S5K Fig). Therefore, reduction of Myc as well as elevation of Myc disrupts margin cell fate during regeneration, underscoring the importance of tightly regulating expression of this pro-regeneration, pro-growth factor. However, this phenotype means that rescuing the *brat*^*1*^*/+* phenotype by reducing Myc is not possible, given that reducing Myc alone disrupts the wing margin, and we cannot rule out the possibility that other Brat targets in addition to Myc contribute to the patterning defects in *brat*^*1*^*/+* regenerated wings. Importantly, animals that overexpressed Myc in the wing pouch during ablation did not undergo a regeneration-induced pupariation delay (S5L Fig), confirming that Brat regulates the entry into metamorphosis independently of its regulation of Myc. Therefore, not all effects of reduced Brat are mediated through Myc.

### Enhanced proliferation does not disrupt margin cell-fate specification during regeneration

Myc is an important driver of regenerative growth, and yet, we found that cell-fate specification during regeneration can be negatively affected if Myc is overexpressed. To test whether the aberrant patterning was a specific result of high Myc levels or whether increases in growth and proliferation could, in general, cause margin defects, we also increased proliferation during regeneration by overexpressing both *cyclinE (cycE)* and *string (stg)*.

**Fig 6:**
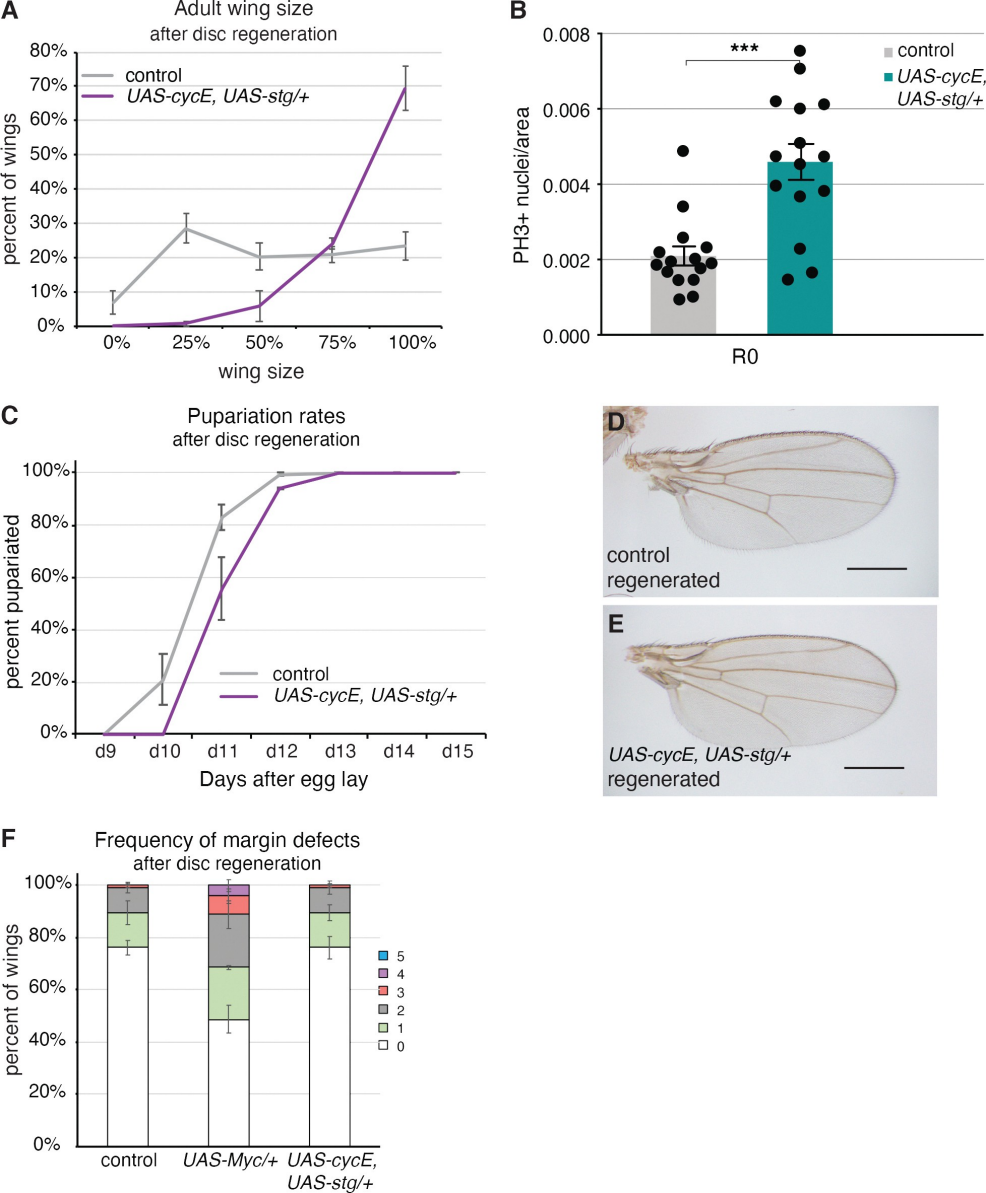
Overexpression of cell cycle genes does not cause patterning defects. (A) Adult wing sizes observed after disc regeneration for control (*w*^*1118*^) (n = 280) and *UAS-cycE*, *UAS-stg/+* (n = 194) wings, from three independent experiments. (B) Pupariation rates after disc regeneration for control (*w*^*1118*^) (n = 174) and *UAS-cycE*, *UAS-stg/+* (n = 115) wings, from three independent experiments. (C) Quantification of mitotic cells positive for anti-PH3 immunostaining in the Nubbin-expressing area of regenerating wings discs at R0. Control n = 15, *UAS-cycE*, *UAS-stg* n = 15. ***p = .0001. (D) Adult control (*w*^*1118*^) wing after disc regeneration. (E) Adult *UAS-cycE*, *UAS-stg/+* wing after disc regeneration. (F) Frequency of margin defects seen in adult wings after disc regeneration for control (*w*^*1118*^) (n = 118), *UAS-Myc/+* (n = 146), and *UAS-cycE*, *UAS-stg/+* (n = 188) wings, from three independent experiments. Error bars represent SEM. Scale bars for adult wings are 0.5 mm.

Overexpressing the cell cycle genes in the wing imaginal disc during the 24-hour ablation period caused the resulting adult wings to be much larger than controls that had also undergone damage and regeneration (Fig 6A), though not larger than a normal wing. Increased proliferation was confirmed through quantification of mitotic cells (Fig 6B). The accompanying increase in pupariation delay appeared less than that induced by reduction of Brat, making the enhanced regeneration even more remarkable (Fig 6C). Intriguingly, we did not observe many margin defects for wings that had experienced *cycE* and *stg* overexpression during regeneration (Fig 6D–6F). Furthermore, we have not observed this loss of margin phenotype with overexpression of other pro-regeneration genes such as *wg* or *ilp8* (S3F and S3G Fig). Thus, pattern disruption during regeneration appears to be specifically associated with Myc overexpression and does not appear to be caused by increased proliferation alone.

### Loss of cell-fate specification may be due to misregulation of Myc target genes

Given that driving growth by overexpressing Cyclin E and String does not cause loss of wing margin cell fates in regenerating tissue, this phenotype might be caused by misregulation of one or more targets of the Myc transcription factor. To identify genes that may regulate margin cell fate downstream of Myc, we screened through genes reported in the literature as regulating *ct* expression, and identified one that was misregulated in *brat*^*1*^*/+* and *UAS-Myc* regenerating discs. We have previously identified the gene *Chronologically inappropriate morphogenesis (chinmo)* as a novel regulator of regeneration [50]. Chinmo is a transcription factor that regulates the balance between a proliferative self-renewal state and a differentiated state in stem cells [75,76]. Recent work has shown that *chinmo* also maintains wing epithelial cells in an unspecified state during development by inhibiting *ct* expression, and enhances regenerative potential [77]. Therefore, we included *chinmo* in our targeted screen for genes that are misregulated in the *brat*^*1*^*/+* or *UAS-Myc* regenerating discs and that contribute to the disruption of cell fate.

**Fig 7:**
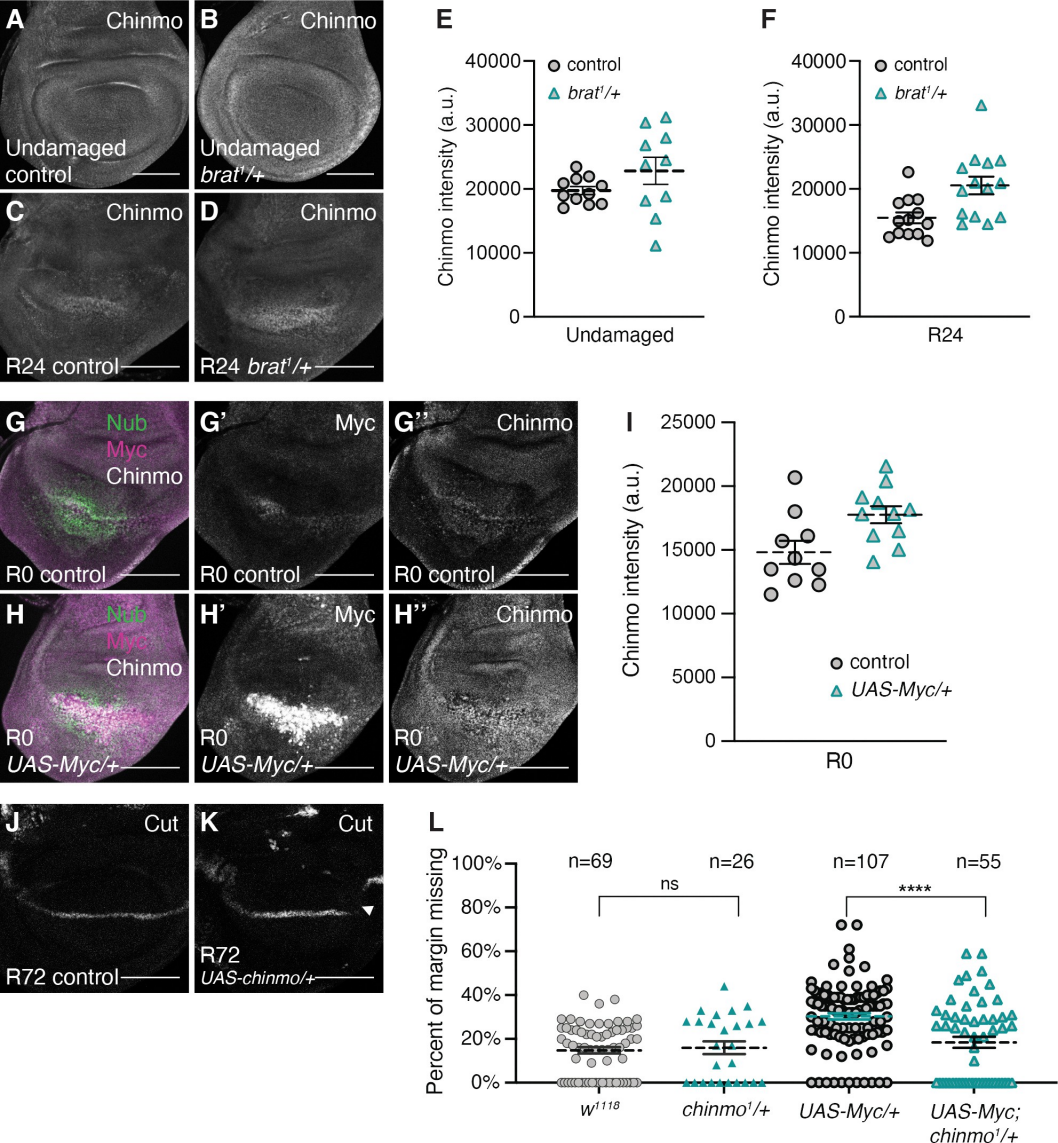
Chinmo levels are elevated in *brat*^*1*^*/+* and Myc-overexpressing regenerating discs. (A-B) Anti-Chinmo immunostaining in an undamaged control (*w*^*1118*^) disc (A) and an undamaged *brat*^*1*^*/+* disc (B). (C-D) Anti-Chinmo immunostaining in an R24 control (*w*^*1118*^) disc (C) and an R24 *brat*^*1*^*/+* disc (D). (E) Quantification of Chinmo fluorescence intensity in undamaged control (*w*^*1118*^) (n = 11) and undamaged *brat*^*1*^*/+* (n = 10) discs. p = 0.16 (F) Quantification of Chinmo fluorescence intensity in R24 control (*w*^*1118*^) (n = 13) and R24 *brat*^*1*^*/+* (n = 14) discs. p < 0.006. Area for fluorescence intensity measurement was defined by the elevated Chinmo expression domain in the wing pouch. (G) Merge of anti-Nubbin, anti-Myc and anti-Chinmo immunostaining in an R0 control (*w*^*1118*^) disc. (G’-G”) Same disc as (G) showing anti-Myc and anti-Chinmo immunostaining, respectively. (H) Merge of anti-Nubbin, anti-Myc and anti-Chinmo immunostaining in an R0 *brat*^*1*^*/+* disc. (H’-H”) Same disc as (H) showing anti-Myc and anti-Chinmo immunostaining, respectively. (I) Quantification of Chinmo fluorescence intensity in R0 control (*w*^*1118*^) (n = 10) and R0 *UAS-Myc/+* (n = 11) discs. p < 0.02. Area for fluorescence intensity measurement was defined by the elevated Myc expression domain in the wing pouch. (J-K) Anti-Cut immunostaining in control (J) and UAS-chinmo (K) R72 discs. Arrowhead shows missing Cut expression in K. (L) Margin tissue lost as a percentage of total wing perimeter for control (*w*^*1118*^) (n = 69), *chinmo*^*1*^*/+ (n = 26)*, *UAS-Myc/+* (n = 107), and *UAS-Myc/+; chinmo*^*1*^*/+* (n = 55) wings. ****p = 9.11E-05. Error bars represent SEM. Student’s T-test used for statistical analyses. Scale bars are 100 μm.

Several lines of evidence suggest that *chinmo* can be a transcriptional target of Myc. First, the model organism Encyclopedia of Regulatory Networks (modERN) data show Myc binding near the *chinmo* promoter [78]. Second, ChIP-seq experiments in *Drosophila* embryonic Kc cells have shown that Myc does indeed bind to the *chinmo* locus [79]. Finally, *chinmo* levels are upregulated in response to elevated Myc expression in wing imaginal discs [80]. To determine whether *chinmo* expression was altered in *brat*^*1*^*/+* wing discs, we immunostained for Chinmo protein. Chinmo levels were not significantly different in undamaged control and *brat*^*1*^*/+* discs (Fig 7A, 7B and 7E). However, Chinmo levels were significantly higher in *brat*^*1*^*/+* regenerating discs compared to control regenerating discs at R24 (Fig 7C, 7D and 7F). To confirm regulation of *chinmo* downstream of Myc, we examined Chinmo levels in regenerating discs over-expressing Myc. Chinmo levels were elevated in Myc-overexpressing discs at R0, when Myc overexpression was the highest (Figs 5F and 7G–7I). However, Chinmo levels were restored to control levels by R24 in Myc-overexpressing discs, consistent with the return of Myc levels to normal at this time point (Figs 5F and S6A–S6C). Interestingly, Myc and Chinmo expression almost perfectly co-localized, consistent with the hypothesis that Myc regulates Chinmo expression (S6A–S6B”’ Fig). Additionally, we observed a high correlation between Myc and Chinmo expression levels in individual discs (S6D and S6E Fig). Undamaged discs overexpressing Myc did not show elevated Chinmo levels (S6F–S6H Fig), possibly explaining why Myc overexpression during normal development did not cause margin defects.

While *chinmo* mRNA can be a direct Brat target [39], *chinmo* is regulated at the level of transcription in the wing imaginal disc [77]. To confirm the absence of regulation of *chinmo* mRNA by Brat during regeneration, we assessed expression of mCherry from a transgene in which the *chinmo* UTRs were fused to the *mCherry* coding region to serve as a reporter for *chinmo* mRNA regulation (*UAS-mCherry-chinmoUTR*) [77]. We did not detect any change in mCherry levels in *brat*^*1*^*/+* regenerating discs compared to control regenerating discs, indicating that reduction of Brat does not impact the *chinmo* mRNA reporter and Brat does not likely regulate *chinmo* mRNA in regenerating discs (S6I Fig).

Thus, the loss of *ct* expression and loss of margin cell fates in *brat/+* regenerating discs and *UAS-Myc* regenerating discs may be due, at least in part, to upregulation of Myc targets, including *chinmo*. Importantly, other unidentified Myc targets likely also contribute to *ct* misregulation in regenerating discs with reduced Brat or overexpressed Myc. To test whether expression of *chinmo* in the regeneration blastema is sufficient to disrupt the margin, we expressed *UAS*-*chinmo* alongside the ablation system. Doing so led to a moderate level of gaps in cut expression in R72 regenerating discs (4/14 compared to 0/12 for controls) (Fig 7J and 7K). To test whether the loss of margin in regenerating wing discs overexpressing Myc is due at least in part to expression of *chinmo*, we asked whether the phenotype could be ameliorated by a *chinmo* mutation. Indeed, regenerating wing discs overexpressing Myc but heterozygous for *chinmo*^*1*^ had significantly less margin loss in the resulting adult wings compared to those just overexpressing Myc (Fig 7L). Thus, we have identified at least one Myc target that contributes to disruption of cell fate in regenerating tissue with Myc expression elevated beyond what is normally found in the regeneration blastema.

We also tested whether reduction of Chinmo expression could partially rescue the *brat* heterozygous phenotype in adult wings and the impaired return of Ct expression at R72. This experiment did not provide a conclusive answer, as it resulted in too few adult wings large enough to quantitate margin defects, likely because *chinmo* heterozygotes regenerate poorly. We were able to quantitate return of Ct expression at R72, and found that reduction of Chinmo led to an increase in the percentage of discs with strong Ct expression, which could suggest a partial rescue, but also an increase in discs with no Ct expression (S6J Fig). The average percentage of the margin that expressed Ct was not statistically different when comparing *brat*^*1*^*/+* to *brat*^*1*^*/+*, *chinmo*^*1*^*/+* R72 discs (S6K Fig). Therefore, reduction of *chinmo* expression did not rescue the *brat*^*1*^*/+* margin defect phentotype as assessed by *cut* expression in the regenerating disc.

**Fig 8:**
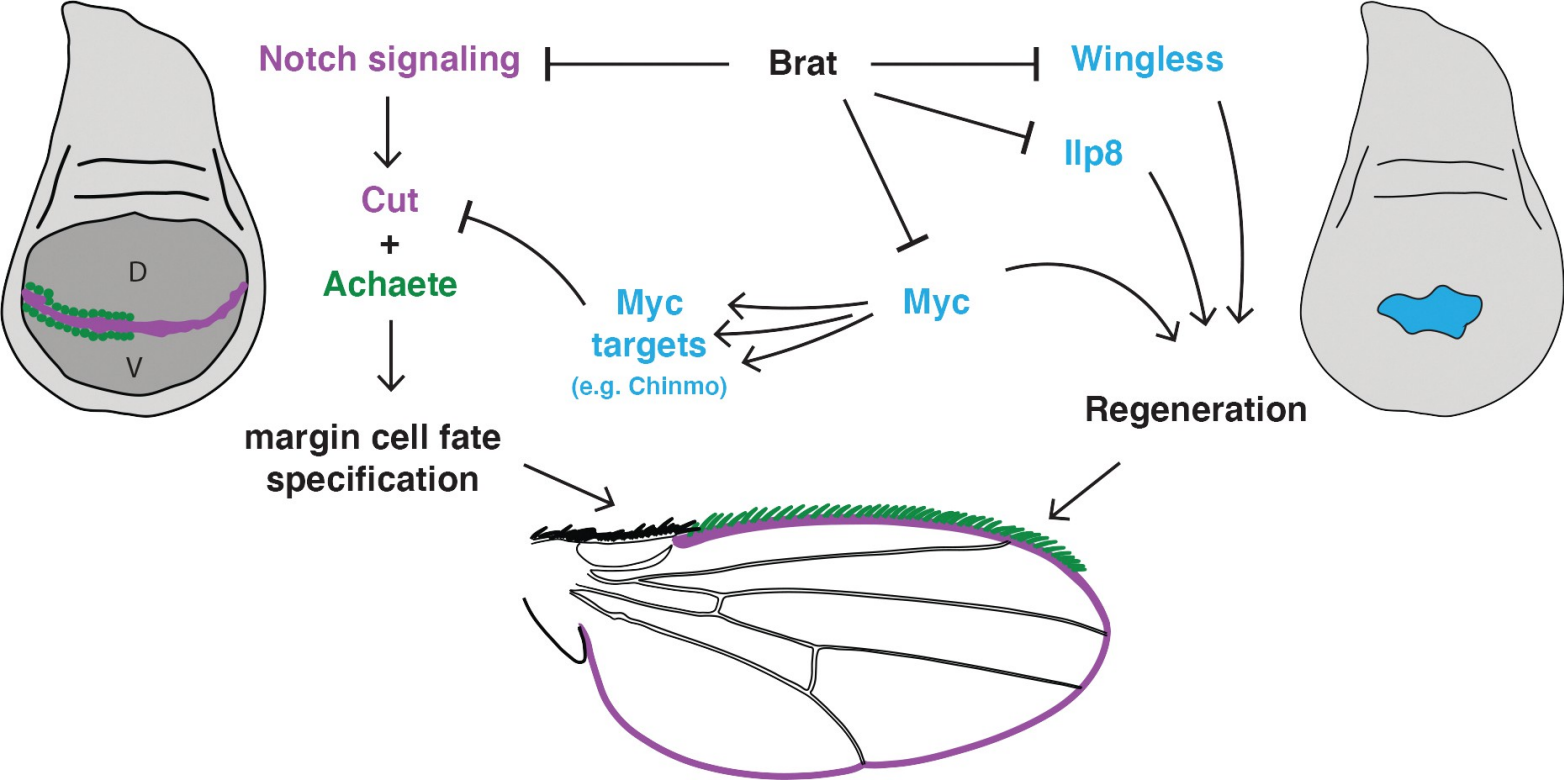
Brat restricts pro-regeneration factors and ensures correct margin cell-fate specification. Model describing the network of Brat targets in the regenerating wing imaginal disc. Importantly, Brat restricts Myc levels, limiting regenerative growth. Elevated Myc levels can result in expression of Myc targets, including Chinmo, which disrupt margin cell-fate specification.

Based on our findings, we propose a model in which pro-growth factors are important for coordinating regenerative growth, but can lead to deleterious side effects by perturbing cell-fate gene expression and patterning. Brat prevents a prolonged proliferative and unspecified state in regenerating wing discs by constraining Wg, Ilp8, Myc, and Myc targets, to enable cessation of growth, induction of cell-fate specification, and entry into metamorphosis (Fig 8).

## Discussion

Here we have shown that Brat constrains regenerative growth at least in part by limiting expression of Wg, Myc, and Ilp8. Wg and Myc are important for driving regenerative growth, and elevated levels enhance regeneration [21,23,25]. Ilp8 expression induces a systemic delay in pupariation, which allows more time for regeneration, and artificially lengthening this delay enhances regeneration [81]. Given that faster and more complete regeneration would be a desired outcome, why does Brat constrain pro-regeneration factors?

Importantly, we have also shown that Brat acts as a protective factor during regeneration, ensuring proper cell fate and patterning along the wing margin. Indeed, Brat prevents wing margin defects both under our genetic screen conditions, in which few controls regenerate to produce full-size wings (Fig 1H and 1I) as well as in experiments where 18% of controls did regenerate well, such as S5I Fig. We propose that these effects occur in part by constraining levels of the transcription factor Myc, which promotes growth and proliferation but also inhibits cell-fate specification. If Brat is unable to perform its protective function during regeneration, Myc levels increase unchecked, resulting in misregulation of its targets, causing loss of proper cell fates at the wing margin.

We have previously noted activity of a signaling circuit in the regenerating tissue in which Wg suppresses N, which suppresses Myc, first described at the wing margin [82]. Brat reduction had an independent effect on Notch signaling, leading to its upregulation concomitant with upregulation of Wingless expression in the *brat* heterozygous mutants. This elevation of N signaling did not suppress Myc expression, perhaps because it was significantly less than that induced by overexpression of intracellular Notch (Figs 4I and S4F). Indeed, a reduction in Myc caused by overexpression of the Notch intracellular domain may account for the improvement in margin pattern in the resulting adult wings (S4G Fig).

Myc is broadly used across organisms to promote proliferation and prevent differentiation [83,84]. It is required for efficient regeneration, and increased levels can enhance regeneration in both younger discs as well as mature discs that normally regenerate poorly [23,25]. Nevertheless, we have found that while abnormally high Myc levels can enhance regenerative growth, they can also perturb differentiation by misregulating target genes such as *chinmo*. Thus, enhanced regeneration happens at the expense of correct cell-fate specification. Intriguingly, our attempts to rescue the *brat/+* phenotype by reducing Myc led to the discovery that loss of Myc expression in the regeneration blastema also leads to loss of margin cell fates. Many Myc targets are ribosomal protein genes, and, interestingly, adults transheterozygous for mutations in the ribosomal protein gene *RpL38* also have missing wing margins even without damage and regeneration of the wing disc [85]. This finding provides precedent for disruption of margin fates when ribosomal protein expression is reduced, and suggests that the reasons for the lost margin in the *Myc*^*P0*^ and *UAS-Myc* regenerating discs may be different. Importantly, expression of *ct* and possibly other cell-fate genes requires an appropriate amount of Myc, with too much or too little Myc disrupting cell fate. This phenomenon of requiring just the right amount of signaling or gene activity is not unprecedented, as we have previously shown that activation of JNK signaling after wing disc damage requires the right amount of reactive oxygen species (ROS) production; too much ROS and too little ROS both lead to inadequate JNK activity and poor regeneration [51].

Brat promotes differentiation in *Drosophila* larval neuroblasts and ovarian germline stem cells by asymmetrically segregating to one of the daughter cells where it post-transcriptionally inhibits Myc [41,42]. This daughter cell is then able to differentiate while the other daughter cell remains a stem cell. In *brat* mutants, progeny of stem cells are unable to differentiate, resulting in an abnormal expansion of the stem-cell population, which can form tumors in the brain [40–43]. Thus, Brat protects these tissues from overproliferation of stem cells. Importantly, wing imaginal disc regeneration is not stem-cell based, but in wing disc regeneration Brat also inhibits Myc to allow correct cell-fate specification. Based on these similarities in function, Brat likely acts as a protective factor across different biological contexts, including regeneration that does not employ stem cells.

We have previously shown that JNK signaling can induce posterior-to-anterior fate changes in regenerating wing discs, which can be prevented by the protective factor Taranis [32]. In addition, we have shown that the chromatin-remodeling SWI/SNF BAP complex, defined by the complex-specific component Osa, similarly prevents aberrant posterior-to-anterior fate changes in regenerating wing discs [86]. We have now identified a third protective factor, Brat, which is needed specifically for correct patterning of the regenerating wing margin. Interestingly, while elevated JNK signaling causes anterior markers to appear in the posterior wing compartment, it does not cause margin loss, indicating that posterior fate and margin fate are regulated in distinct ways [32]. Protective factors such as Tara, Osa, and Brat are important for maintaining the balance between fate specification and regenerative potential, but they do so by using very different mechanisms. While the molecular function of Tara is unknown, genetic interactions in *Drosophila* coupled with the demonstrated functions of its vertebrate homologs suggest it may regulate gene expression at the level of transcription and chromatin [87–90]. By contrast, Brat acts as a translational repressor by negatively regulating translation and stability of its target mRNAs [91,92]. Tara is required to prevent fate changes induced by JNK signaling, which is necessary for wound repair and regeneration but is not required for the normal growth of the wing. By contrast, Myc is required for both development and regeneration of the wing disc, but is constrained by Brat only during regeneration.

An important open question in the field of regeneration is how patterning and cell-fate specification are regulated in regenerating tissue, and whether these mechanisms are different from the developmental program. Many studies have highlighted that regeneration must be distinct from development in some ways, because the damaged tissue already has complex patterning, and the wound-healing response causes strong activation of signaling pathways, some of which are not normally present in developing tissue [1,3,25,30–35]. We are just beginning to identify regulators like Brat that are crucial for attenuating regenerative growth signaling and shielding the regenerating tissue from the harmful side effects of such signaling. Identification of these regulators highlights the fact that the regenerating tissue is distinct from normally developing tissue. Since regeneration signaling is complex and comprised of many signaling pathways, additional factors that play protective roles during regeneration likely exist. Identification of these factors will be important for the development of clinical therapies targeted at tissue repair, enabling these therapies to protect against the deleterious side effects of exogenous and unconstrained pro-growth signaling.

## Materials and methods

### Ablation and regeneration experiments

Ablation experiments were done as previously described [32]. Briefly, cell death was induced by driving *UAS-reaper* under *rotund-GAL4*, with *GAL80*^*ts*^ for temporal control. Eggs were collected over the course of four hours and animals were raised at 18°C for 7 days after egg lay (AEL) (early third instar as determined by mouth hooks) before they were shifted to a 30°C circulating water bath for 24 hours. Animals were brought back to 18°C to allow regeneration. Wing discs were dissected at different time points after the end of ablation, or the animals were allowed to grow to adulthood to observe the adult wing phenotype. Undamaged control wing discs were the same genotype as the experimental animals but kept at 18°C and dissected on day 9 after egg lay, which is mid-late third instar. For undamaged adult wings, the animals were kept at 18°C until after eclosion. Any other undamaged conditions used are mentioned specifically in the Fig legends.

### Fly stocks

The following *Drosophila* stocks were used: *w*^*1118*^ (wild type) [93], *w*^*1118*^*; rnGAL4*, *UAS-rpr*, *tubGAL80ts/TM6B*,*tubGAL80* [23], *brat*^*1*^ [52](RRID:BDSC_3988), *brat*^*192*^ and *brat*^*150*^ [54](a gift from Juergen Knoblich, Austrain Academy of Science), *brat*^*11*^ [55](a gift from Chen-Yu Lee, University of Michigan), *Df(2L)Exel8040* [94](RRID:BDSC_7847), *Df(2L)TE37C-7* [53](RRID:BDSC_6089), *rnGAL4*, *tubGAL80ts/TM6B* [23], *P{Trip*.*HM05078}attP2* (called *bratRNAi* in the text)(RRID:BDSC_28590), *P{CaryP}attP2* (called *attP2* control in the text)(RRID:BDSC_36303), *{PZ}ap*^*rK568*^ [95](RRID:BDSC_5374), *NRE-GFP* [68](RRID:BDSC_30727), *UAS-Nintra* (a gift from Gary Struhl, Columbia University), *aph-1*^*D35*^ [96](RRID:BDSC_63242), *UAS-Myc* [74](RRID:BDSC_9674), *UAS-cycE*, *UAS-stg* (a gift from Laura Buttitta, University of Michigan), *dm*^*4*^ [72], *P{GD1419}v2947* (called *MycRNAi#1* in the text)(VDRC ID# 2947) and *P{GD1419}v2948* (called *MycRNAi#2* in the text)(VDRC ID# 2948), *P{GD6000}v15293* (called control in the text) (VDRC ID# 15293), *Myc*^*P0*^ [74] (RRID:BDSC_11298), UAS-mCherry^chinmoUTRs^ [75,76], *chinmo*^*1*^ [97] (RRID:BDSC_59969), UAS-*wg* (RRID:BDSC_5918), UAD-*dilp8* [59](a gift from Maria Dominguez). All fly stocks are available from the Bloomington Drosophila Stock Center unless stated otherwise.

### Pupariation timing

Pupariation experiments were performed in a similar manner to the ablation experiments. Starting at day 9, newly formed pupal cases were counted in each vial. Pupal cases were counted every 24 hours, up until day 15. Pupariation rates from three independent experiments were used to calculate the average plotted in the graphs.

### Immunohistochemistry

Immunostaining was carried out as previously described [23], with a single batch of antibody diluted in buffer generated for each experiment to ensure equal concentrations across samples for which immunostaining was quantified. Primary antibodies were rat anti-Brat (1:200) [37] (a gift from Robin Wharton, Ohio State University), mouse anti-Nubbin (1:500) [98] (a gift from Steve Cohen, University of Copenhagen) and 1:100 (The Developmental Studies Hybridoma Bank [DSHB]), rabbit anti-Phospho-Histone H3 (1:500) (Millipore), mouse anti-Wingless (1:100) (DSHB), rabbit anti-dMyc (1:500 or 1:250) (Santa Cruz Biotechnologies), mouse anti-βgal (1:100) (DSHB), mouse anti-Cut (1:100) (DSHB), mouse anti-Achaete (1:10)(DSHB), rat anti-Chinmo (1:500) (a gift from Nick Sokol, Indiana University), and anti-cleaved Dcp-1 (1:250)(Cell Signaling #9578). The Developmental Studies Hybridoma Bank (DSHB) was created by the NICHD of the NIH and is maintained at the University of Iowa, Department of Biology, Iowa City, IA 52242.

Secondary antibodies were AlexaFluor probes (1:1000) (Life Technologies). DNA was marked using TO-PRO3 (1:500) (Life Technologies) or DAPI (1:5000 of 0.5 mg/mL stock) (Sigma). Discs were mounted in Vectashield mounting medium (Vector Laboratories).

EdU incorporation was carried out as previously described [99] using a Click-iT EdU kit C10338 (Invitrogen), with the following changes. Larvae were dissected in PBS and incubated in Schnieder’s medium. EdU was used at a working concentarion of 100 μM.

Discs were imaged on a Zeiss LSM 510, Zeiss LSM 700, or a Zeiss LSM 880 confocal microscope. Parameters for imaging were identical for quantified images. Images were processed using ZEN lite (Zeiss), ImageJ (NIH) and Photoshop (Adobe). Maximum intensity projections were created for the confocal images. Fluorescence intensity was measured within the wing pouch as marked by anti-Nubbin or by using the morphology of the undamaged wing disc. Myc and Chinmo intensities were measured by outlining the region expressing elevated Myc or Chinmo levels. *NRE-GFP* intensity was measured by outlining the GFP-expressing region at the DV boundary. Any modifications to image bightness and contrast to assist visibility for publication were applied equally to all images from the same experiment.

### Adult wing quantifications

Adult wings were mounted in Gary’s Magic Mount (Canada balsam [Sigma] dissolved in methyl salicylate [Sigma]). Images were taken with an Olympus SZX10 microscope with an Olympus DP21 camera using the CellSens Dimension software (Olympus) or with an Echo Revolve R4 microscope and camera.

All adult wings that were 75% or 100% the size of a normal wing were used to quantify the loss of the wing margin. The wing margin was divided into five segments defined by where the wing veins intersect the margin. Each wing was scored for the number of segments with missing margin to assess the extent of the patterning defect. Percentages from the three independent experiments were used to calculate averages plotted in the graphs. The area of undamaged and regenerated wings was measured using ImageJ (NIH). ImageJ was also used to measure the percentage of linear length of margin lost for the entire perimeter of the wing. Graphs were plotted using Excel and Graphpad Prism 7.

### qPCR

For quantitative PCR (qPCR), 40–60 wing imaginal discs were collected in Schneider’s medium and stored at -80°C. RNA was extracted using the Qiagen RNeasy Mini Kit (#74104), and cDNA synthesis was performed using the Superscript III First Strand Synthesis kit (#11752–050). qPCR reactions using the Power SYBR Green MasterMix (ABI) were run on the ABI Step One Plus Real Time PCR System. The experiment consisted of 3 biological replicates. For each biological replicate there were three technical replicates. Gene expression was analyzed by the ΔΔC_t_ method and normalized to *Gapdh2* expression. The following primers were used: *Gapdh2* forward primer (GTGAAGCTGATCTCTTGGTACGAC), *Gapdh2* reverse primer (CCGCGCCCTAATCTTTAACTTTTAC) [100], *ilp8* primers used from Qiagen (QT00510552), *dmyc* forward primer (AACGATATGGTGGACGATGG), and *dmyc* reverse primer (CGGCAGATTGAAGTTATTGTAGC) [101]. For qPCR experiments undamaged controls were *rnGAL4*, *tubGAL80ts/TM6B* females crossed to *w*^*1118*^ males and shifted to 30°C for 24 hours at 7 days AEL. Discs were dissected either immediately or 24 hours after shifting the animals back to 18°C for R0 and R24 time points, respectively.

## Supporting information

S1 Fig Reduction of *brat* expression does not cause enhanced growth or margin defects during normal development (Related to Fig 1). (A) Adult wing area measured using ImageJ after mounting and imaging wings, for undamaged control (*w*^*1118*^) (n = 63 female and 70 male) and *brat*^*1*^*/+* (n = 38 female and 48 male) wings. *rnGAL4*, *GAL80*^*ts*^*/TM6B* females were crossed to *w*^*1118*^ or *brat*^*1*^*/SM6-TM6B* males and taken through the protocol shown in Fig 1A. Differences in size are extremely small though statistically significant (p = 0.01 for females and 2.15x10^-14^ for males). (B) Adult wing sizes after disc regeneration for control (*w*^*1118*^) (n = 599), *brat*^*1*^*/+* (n = 199), *brat*^*192*^*/+* (n = 237), *brat*^*150*^*/+* (n = 235) and *brat*^*11*^*/+* (n = 188) wings, from three independent experiments. (C) Margin defects detected in adult wings from undamaged control (*w*^*1118*^) and *brat*^*1*^*/+* discs. *rnGAL4*, *GAL80*^*ts*^*/TM6B* females were crossed to *w*^*1118*^ or *brat*^*1*^*/SM6-TM6B* males and taken through the protocol shown in Fig 1A. Margin defects detected in the undamaged wings were never as severe as the ones seen in *brat*^*1*^*/+* wings after disc regeneration. A representative wing with margin defects is shown. (D) Anti-Brat immunostaining in undamaged control (*attP2*) and *bratRNAi/+* discs. *rnGAL4*, *GAL80*^*ts*^*/TM6B* females were crossed to *attP2* or *bratRNAi* males. Larvae were kept at 18°C and shifted to 30°C on day 7 AEL. Discs were dissected 24 hours after the shift to 30°C. Quantification of Brat fluorescence intensity in undamaged control *(attP2*) (n = 15) and *bratRNAi*/+ (n = 15) discs. Area for fluorescence intensity measurement was defined by wing pouch morphology and Anti-Myc co-immunostaining. * p = 0.02. (E) Margin defects detected in adult wings from undamaged control (*attP2*) and *bratRNAi* dics. *rnGAL4*, *GAL80*^*ts*^*/TM6B* females were crossed to *attP2* or *bratRNAi* males. Larvae were kept at 18°C and shifted to 30°C on day 7 AEL and kept there until eclosion. (F) Frequency of margin defects seen in adult wings after disc regeneration for control (*w*^*1118*^) (n = 240), *brat*^*1*^*/+* (n = 191), *brat*^*192*^*/+* (n = 196), *brat*^*150*^*/+* (n = 213) and *brat*^*11*^*/+* (n = 152) wings. Wings in (F) are from the same experiments as (B). (G) Quantification of fluorescence intensity of anti-Brat immunostaining at R0 in control (n = 10) and *UAS-bratRNAi* (n = 5) discs showing no reduction of protein levels. Error bars represent SEM. Student’s T-test used for statistical analyses. Scale bars are 100 μm. Scale bars for adult wings are 0.5 mm.
(PDF)

S2 Fig Reduction of *brat* expression delays pupariation in a regeneration-specific manner (Related to Fig 2). (A) Pupariation rates in undamaged control (*w*^*1118*^) (n = 221) and *brat*^*1*^*/+* (n = 110) animals, from three independent experiments. (B) Pupariation rates after disc regeneration for control (*w*^*1118*^) (n = 384), *brat*^*1*^*/+* (n = 107), *brat*^*192*^*/+* (n = 131), *brat*^*150*^*/+* (n = 114) and *brat*^*11*^*/+* (n = 113) animals. Pupariation rates are from the same experiments as in Fig 2A. (C) Nubbin-positive nuclei were counted at R0 in control regenerating (n = 12) and *brat*^*1*^*/+* regenerating (n = 11) discs. (D) PH3-positive nuclei were counted within the regenerating tissue as marked by Anti-Nubbin co-immunostaining. Total number of nuclei were also counted in the Nubbin expressing region. Ratio of PH3-positive nuclei and total Nubbin-positive nuclei for control (*w*^*1118*^) and *brat*^*1*^*/+* discs at R0 (n = 16 and 18). (E) EdU incorporation marking DNA synthesis in cells in S phase was quantified by normalizing pixel intensity in the regenerating pouch to pixel intensity in the notum. Control n = 7 discs, *brat*^*1*^*/+* n = 5 discs. *p<0.05. *** p < 0.0005. Error bars represent SEM. Student’s T-test used for statistical analyses. Error bars represent SEM.
(PDF)

S3 Fig Effects of reduced Brat on Wg and Myc expression are regeneration specific (Related to Fig 3). (A-B) Anti-Wg immunostaining in an undamaged control (*w*^*1118*^) disc (A) and an undamaged *brat*^*1*^*/+* disc (B). (C-D) Anti-Myc immunostaining in an undamaged control (*w*^*1118*^) disc (C) and an undamaged *brat*^*1*^*/+* disc (D). (E) Quantification of Myc fluorescence intensity in undamaged control (*w*^*1118*^) (n = 10) and *brat*^*1*^*/+* (n = 10) discs. Area for fluorescence intensity measurement was defined by wing pouch morphology and the elevated Myc expression domain in the wing pouch. Error bars represent SEM. (F) Representative adult wing after expression of *UAS-wg* during regeneration. (G) Representative adult wing after expression of *UAS-ilp8* during regeneration. Student’s T-test used for statistical analyses. Scale bars are 100 μm for imaginal discs and 500 μm for adult wings.
(PDF)

S4 Fig Elevated Notch signaling does not cause margin defects (Related to Fig 4). (A-B) *ap-lacZ* expression in an R72 control (*w*^*1118*^) disc (A) and an R72 *brat*^*1*^*/+* disc (B). Dashed yellow lines are drawn next to the DV boundary to highlight it. (C-D) Anti-Wg (green) and anti-cleaved Dcp1 (magenta, gray in C’ and D’) immunostaining in an R48 control (*w*^*1118*^) disc (C, C’) and an R48 *brat*^*1*^*/+* disc (D, D’). (E-F) *NRE-GFP* expression in an R24 control (*w*^*1118*^) disc (E) and an R24 *UAS-Nintra/+* disc (F). (G) Frequency of margin defects seen in adult wings after disc regeneration for control (*w*^*1118*^) (n = 84) and *UAS-Nintra/+* (n = 357) wings, from five independent experiments. (H-I) *NRE-GFP* expression in an undamaged control (*w*^*1118*^) disc (H) and an undamaged *aph-1*^*D35*^*/+* disc (I). *NRE-GFP*/+ and *NRE-GFP*/*aph-1*^*D35*^ animals were raised at room temperature and dissected during third instar. (J) Quantification of GFP intensity in undamaged control (*w*^*1118*^) (n = 15) and *aph-1*^*D35*^*/+* (n = 15) discs. *** p < 0.0006. (K-L) *NRE-GFP* expression in an R24 control (*w*^*1118*^) disc (K) and an R24 *aph-1*^*D35*^*/+* disc (L). (M) Quantification of GFP intensity in R24 control (*w*^*1118*^) (n = 13) and R24 *aph-1*^*D35*^*/+* (n = 11) discs. * p < 0.02. (N) Frequency of margin defects in adult wings after disc regeneration for control (*w*^*1118*^) (n = 21), *brat*^*1*^*/+* (n = 137), *aph-1*^*D35*^*/+* (n = 38) and *brat*^*1*^*/aph-1*^*D35*^(n = 80) wings. Error bars represent SEM. Student’s T-test used for statistical analyses. Scale bars are 100 μm.
(PDF)

S5 Fig Reduction of Myc expression during regeneration also disrupts margin fate specification (Related to Fig 5). (A) Margin defects detected in adult wings from undamaged control (*w*^*1118*^) and *UAS-Myc/+* discs. *rnGAL4*, *GAL80*^*ts*^*/TM6B* females were crossed to *w*^*1118*^ or *UAS-Myc* males and taken through the protocol shown in Fig 1A. (B) Frequency of margin defects in adult wings after disc regeneration for control (*w*^*1118*^) (n = 103), *brat*^*1*^*/+* (n = 203), *dm*^*4*^*/+* (n = 94) and *dm*^*4*^*/+; brat*^*1*^*/+* (n = 94) wings, from three independent experiments. (C) Quantification of Myc fluorescence intensity in undamaged control (*w*^*1118*^) (n = 12) and *dm*^*4*^*/+* (n = 11) discs. *w*^*1118*^ males were crossed to *w*^*1118*^ or *dm*^*4*^*/FM7i*, *ActGFP* females and dissected when the animals were third instar. Area for fluorescence intensity measurement was defined by wing pouch morphology and the elevated Myc expression domain in the wing pouch. (D) Quantification of Myc fluorescence intensity in R0 control (*w*^*1118*^) (n = 13), R0 *dm*^*4*^*/+* (n = 10), R24 control (*w*^*1118*^) (n = 13), and R24 *dm*^*4*^*/+* (n = 10) discs. Area for fluorescence intensity measurement was defined by the elevated Myc expression domain in the wing pouch. (E) Quantification of Myc fluorescence intensity in undamaged control (VDRC genetic background line, called control) (n = 14), *MycRNAi#1/+* (n = 12), and *MycRNAi#2/+* (n = 13) discs. *rnGAL4*, *GAL80*^*ts*^*/TM6B* females were crossed to the control, *MycRNAi#1*, or *MycRNAi#2* males. The animals were shifted to 30°C during early third instar and kept there for 28 hours then dissected. *MycRNAi#1/+* *** p < 0.000007, *MycRNAi#2/+* *** p < 0.00002. Area for fluorescence intensity measurement was defined by wing pouch morphology. (F) Quantification of Myc fluorescence intensity in R0 control (n = 13), R0 *MycRNAi#1/+* (n = 15), R0 *MycRNAi#2/+* (n = 13), R24 control (n = 13), R24 *MycRNAi#1/+* (n = 13), and R24 *MycRNAi#2/+* (n = 13) discs. Fluorescence intensity was measured in the area marked by Anti-Nubbin immunostaining. *** p < 0.00007. (G,H) Anti-Myc immunostaining in undamaged (G) and regenerating R24 (H) *Myc*^*P0*^*/Y* imaginal wing discs. Dashed line outlines the wing pouch defined by anti-Nubbin immunostaining. (I) Margin tissue lost as a percentage of total wing perimeter for adult wings after disc damage and regeneration in the noted genotypes. (J, K) Immunostaining for Cut in control (J) and *Myc*^*P0*^*/Y* (K) regenerated (R72) discs showing margin disruption in the mutants.(L) Pupariation rates after disc regeneration for control (*w*^*1118*^) (n = 216), *brat*^*1*^*/+* (n = 114) and *UAS-Myc/+* (n = 209) animals, from three independent experiments. Error bars represent SEM. Student’s T-test used for statistical analyses.
(PDF)

S6 Fig Myc regulates Chinmo expression (Related to Fig 7). (A) Merge of anti-Nubbin, anti-Myc and anti-Chinmo immunostaining in an R24 control (*w*^*1118*^) disc. (A’-A”) Same disc as (A) showing anti-Myc and anti-Chinmo immunostaining, respectively. (A”’) Same disc as (A) showing an enlarged merge of anti-Myc and anti-Chinmo immunostaining. (B) Merge of anti-Nubbin, anti-Myc and anti-Chinmo immunostaining in an R24 *UAS-Myc/+* disc. (B’-B”) Same disc as (B) showing anti-Myc and anti-Chinmo immunostaining, respectively. (B”’) Same disc as (B) showing an enlarged merge of anti-Myc and anti-Chinmo immunostaining. (C) Quantification of Chinmo and Myc fluorescence intensity in R24 control (*w*^*1118*^) (n = 13) and R24 *UAS-Myc/+* (n = 14) discs. Area for fluorescence intensity measurement was defined by the elevated Myc expression domain in the wing pouch. Note that Myc and Chinmo expression co-localize. (D-E) Scatter plot showing correlation between Myc and Chinmo expression levels at R0 (D) and R24 (E). Pearson correlation coefficient for R0 = 0.93 and R24 = 0.94. (F) Quantification of Chinmo fluorescence intensity in undamaged discs dissected 31 hours after animals were shifted to 30°C on day 7 AEL. Control (*+; rnGAL4*,*GAL80ts/+*) (n = 14) and *UAS-Myc (UAS-Myc/+; rnGAL4*, *GAL80ts/+)* (n = 14). Area for fluorescence intensity measurement was defined by the Myc expression domain in the wing pouch. (G-H) Anti-Chinmo immunostaining in an undamaged control disc (G) and an undamaged *UAS-Myc/+* disc (H). (I) Quantification of fluorescence intensity from the *UAS-mCherry-chinmoUTR* transgene in control (n = 16) and brat/+ (n = 15) R24 regenerating discs. Area for fluorescence intensity measurement was defined by Nubbin expression. (J) Quantification of wing discs with Ct expression patterns at R72 categorized as complete, almost complete, about half, almost absent, or absent in *brat*^*1*^*/+* (n = 10), *brat*^*1*^*/+*, *chinmo*^*1*^*/+* (n = 17), control *w*^*1118*^ (n = 10), and *chinmo*^*1*^*/+* (n = 8) regenerating discs. (K) Quantification of the fraction of each disc margin that expressed Ct. Genotypes and n same as in J. Error bars represent SEM. Student’s T-test used for statistical analyses. Scale bars are 100 μm, unless stated otherwise.
(PDF)
