# Supplementary figures and images for: Regenerative growth is constrained by brain tumor to ensure proper patterning in Drosophila
Source: PLoS Genet. 2023 Dec 21;19(12):e1011103. doi: 10.1371/journal.pgen.1011103 (PMC10769103; doi:10.1371/journal.pgen.1011103)

**Figure S1**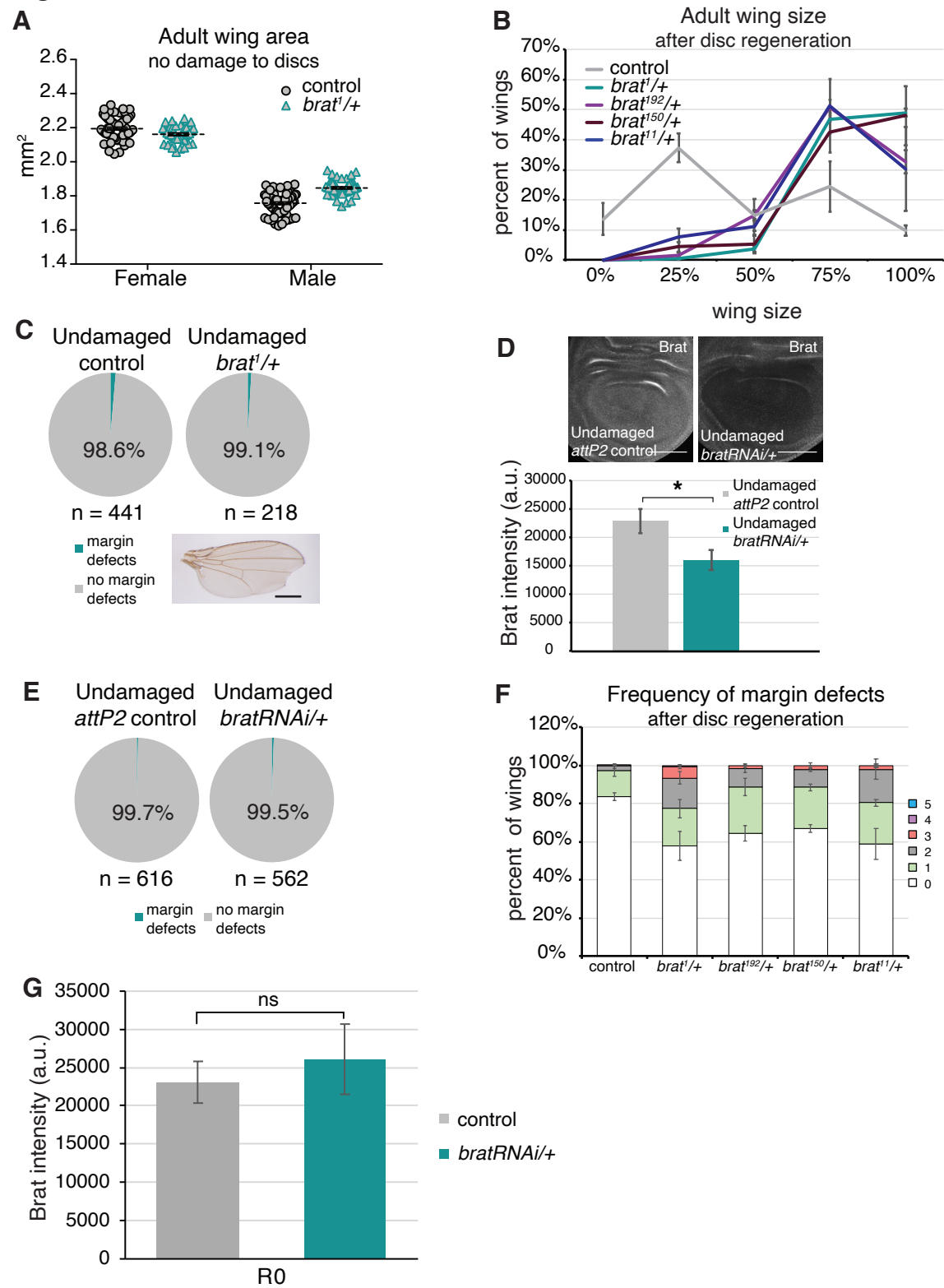

Supplement: S1 Fig — (A) Adult wing area measured using ImageJ after mounting and imaging wings, for undamaged control (w1118) (n = 63 female and 70 male) and brat1/+ (n = 38 female and 48 male) wings. rnGAL4, GAL80ts/TM6B females were crossed to w1118 or brat1/SM6-TM6B males and taken through the protocol shown in Fig 1A. Differences in size are extremely small though statistically significant (p = 0.01 for females and 2.15x10^-14^ for males). (B) Adult wing sizes after disc regeneration for control (w1118) (n = 599), brat1/+ (n = 199), brat192/+ (n = 237), brat150/+ (n = 235) and brat11/+ (n = 188) wings, from three independent experiments. (C) Margin defects detected in adult wings from undamaged control (w1118) and brat1/+ discs. rnGAL4, GAL80ts/TM6B females were crossed to w1118 or brat1/SM6-TM6B males and taken through the protocol shown in Fig 1A. Margin defects detected in the undamaged wings were never as severe as the ones seen in brat1/+ wings after disc regeneration. A representative wing with margin defects is shown. (D) Anti-Brat immunostaining in undamaged control (attP2) and bratRNAi/+ discs. rnGAL4, GAL80ts/TM6B females were crossed to attP2 or bratRNAi males. Larvae were kept at 18°C and shifted to 30°C on day 7 AEL. Discs were dissected 24 hours after the shift to 30°C. Quantification of Brat fluorescence intensity in undamaged control (attP2) (n = 15) and bratRNAi/+ (n = 15) discs. Area for fluorescence intensity measurement was defined by wing pouch morphology and Anti-Myc co-immunostaining. * p = 0.02. (E) Margin defects detected in adult wings from undamaged control (attP2) and bratRNAi dics. rnGAL4, GAL80ts/TM6B females were crossed to attP2 or bratRNAi males. Larvae were kept at 18°C and shifted to 30°C on day 7 AEL and kept there until eclosion. (F) Frequency of margin defects seen in adult wings after disc regeneration for control (w1118) (n = 240), brat1/+ (n = 191), brat192/+ (n = 196), brat150/+ (n = 213) and brat11/+ (n = 152) wings. Wings in (F) are from th [file pgen.1011103.s001.pdf]

**Figure S2**

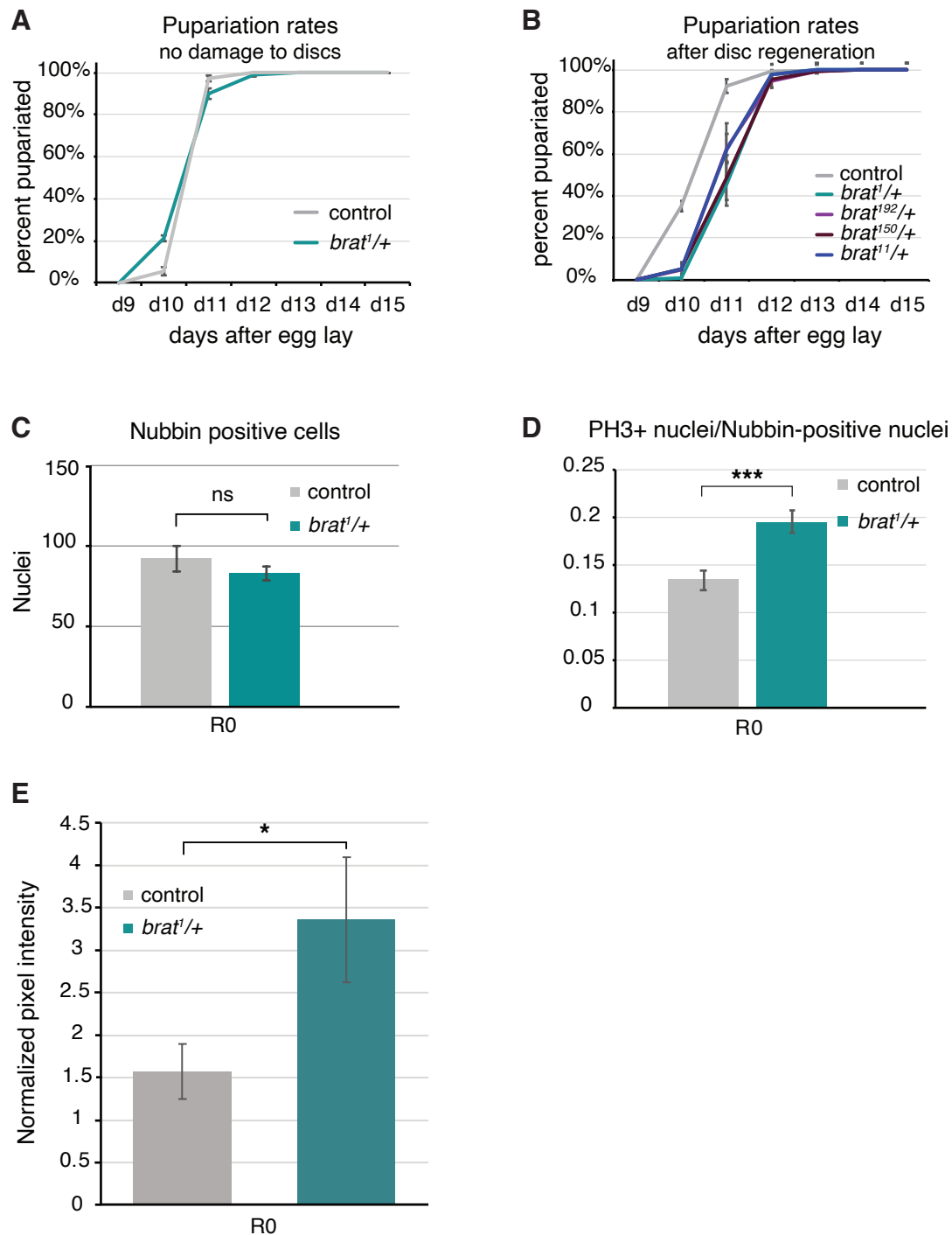

Supplement: S2 Fig — (A) Pupariation rates in undamaged control (w1118) (n = 221) and brat1/+ (n = 110) animals, from three independent experiments. (B) Pupariation rates after disc regeneration for control (w1118) (n = 384), brat1/+ (n = 107), brat192/+ (n = 131), brat150/+ (n = 114) and brat11/+ (n = 113) animals. Pupariation rates are from the same experiments as in Fig 2A. (C) Nubbin-positive nuclei were counted at R0 in control regenerating (n = 12) and brat1/+ regenerating (n = 11) discs. (D) PH3-positive nuclei were counted within the regenerating tissue as marked by Anti-Nubbin co-immunostaining. Total number of nuclei were also counted in the Nubbin expressing region. Ratio of PH3-positive nuclei and total Nubbin-positive nuclei for control (w1118) and brat1/+ discs at R0 (n = 16 and 18). (E) EdU incorporation marking DNA synthesis in cells in S phase was quantified by normalizing pixel intensity in the regenerating pouch to pixel intensity in the notum. Control n = 7 discs, brat1/+ n = 5 discs. *p<0.05. *** p < 0.0005. Error bars represent SEM. Student’s T-test used for statistical analyses. Error bars represent SEM. (PDF) [file pgen.1011103.s002.pdf]

Figure S3

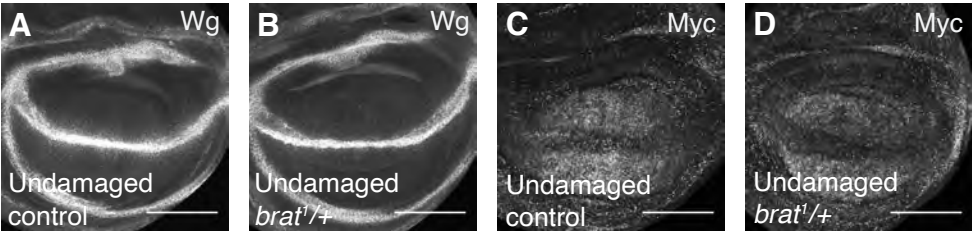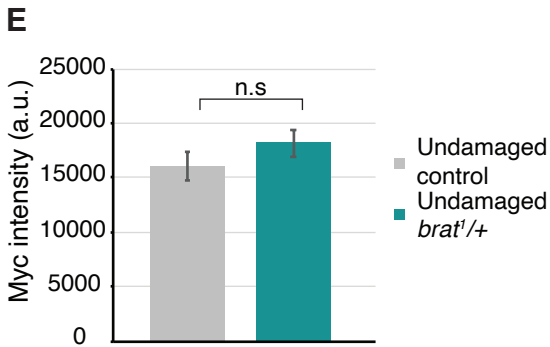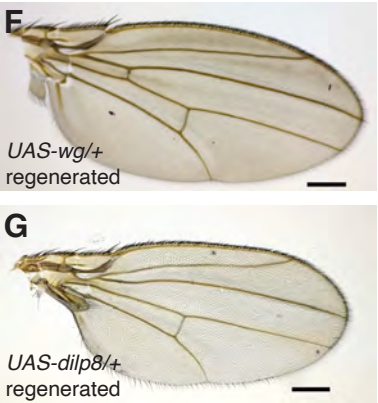

Supplement: S3 Fig — (A-B) Anti-Wg immunostaining in an undamaged control (w1118) disc (A) and an undamaged brat1/+ disc (B). (C-D) Anti-Myc immunostaining in an undamaged control (w1118) disc (C) and an undamaged brat1/+ disc (D). (E) Quantification of Myc fluorescence intensity in undamaged control (w1118) (n = 10) and brat1/+ (n = 10) discs. Area for fluorescence intensity measurement was defined by wing pouch morphology and the elevated Myc expression domain in the wing pouch. Error bars represent SEM. (F) Representative adult wing after expression of UAS-wg during regeneration. (G) Representative adult wing after expression of UAS-ilp8 during regeneration. Student’s T-test used for statistical analyses. Scale bars are 100 μm for imaginal discs and 500 μm for adult wings. (PDF) [file pgen.1011103.s003.pdf]

**Figure S4**

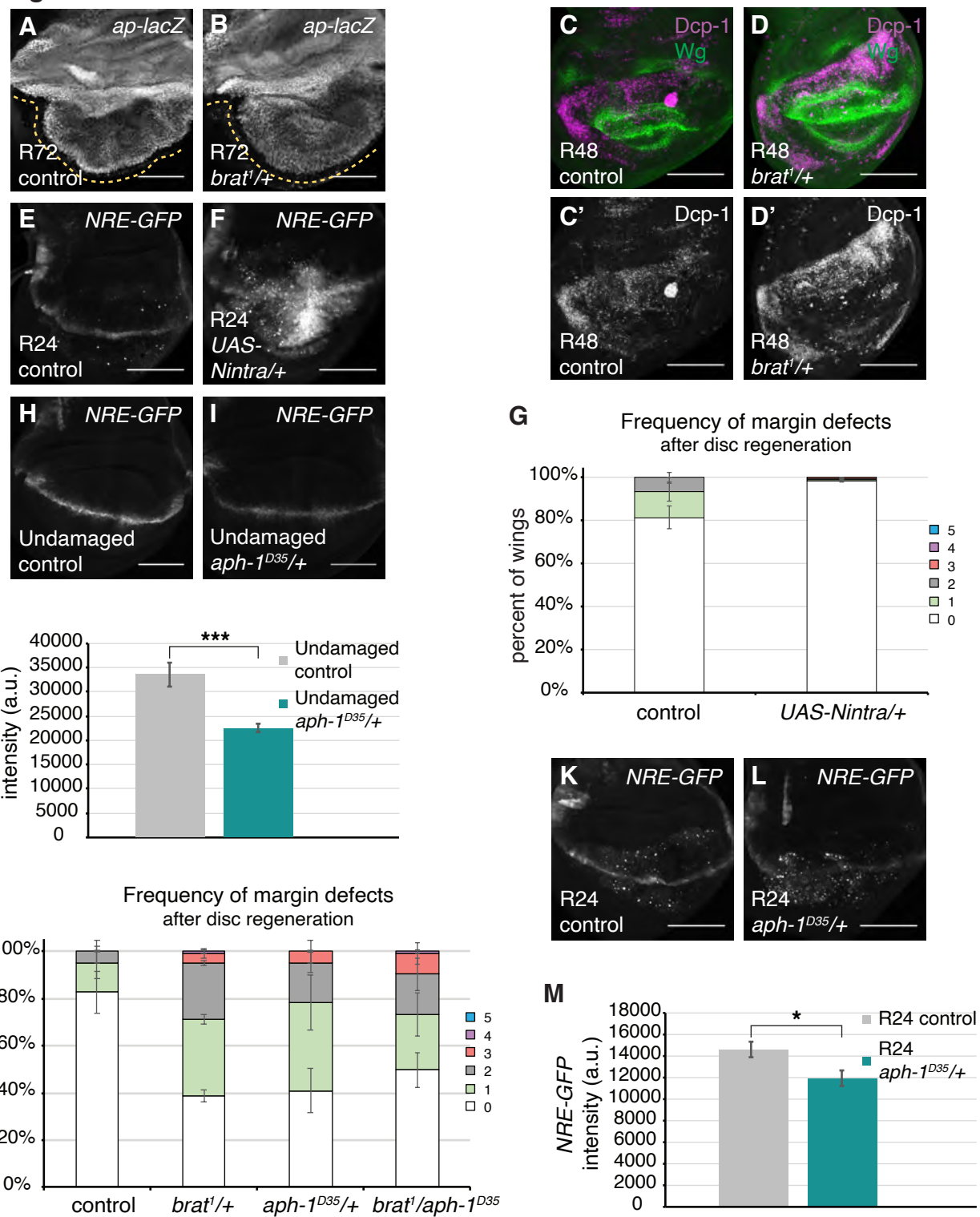

Supplement: S4 Fig — (A-B) ap-lacZ expression in an R72 control (w1118) disc (A) and an R72 brat1/+ disc (B). Dashed yellow lines are drawn next to the DV boundary to highlight it. (C-D) Anti-Wg (green) and anti-cleaved Dcp1 (magenta, gray in C’ and D’) immunostaining in an R48 control (w1118) disc (C, C’) and an R48 brat1/+ disc (D, D’). (E-F) NRE-GFP expression in an R24 control (w1118) disc (E) and an R24 UAS-Nintra/+ disc (F). (G) Frequency of margin defects seen in adult wings after disc regeneration for control (w1118) (n = 84) and UAS-Nintra/+ (n = 357) wings, from five independent experiments. (H-I) NRE-GFP expression in an undamaged control (w1118) disc (H) and an undamaged aph-1D35/+ disc (I). NRE-GFP/+ and NRE-GFP/aph-1D35 animals were raised at room temperature and dissected during third instar. (J) Quantification of GFP intensity in undamaged control (w1118) (n = 15) and aph-1D35/+ (n = 15) discs. *** p < 0.0006. (K-L) NRE-GFP expression in an R24 control (w1118) disc (K) and an R24 aph-1D35/+ disc (L). (M) Quantification of GFP intensity in R24 control (w1118) (n = 13) and R24 aph-1D35/+ (n = 11) discs. * p < 0.02. (N) Frequency of margin defects in adult wings after disc regeneration for control (w1118) (n = 21), brat1/+ (n = 137), aph-1D35/+ (n = 38) and brat1/aph-1D35(n = 80) wings. Error bars represent SEM. Student’s T-test used for statistical analyses. Scale bars are 100 μm. (PDF) [file pgen.1011103.s004.pdf]

**Figure S5**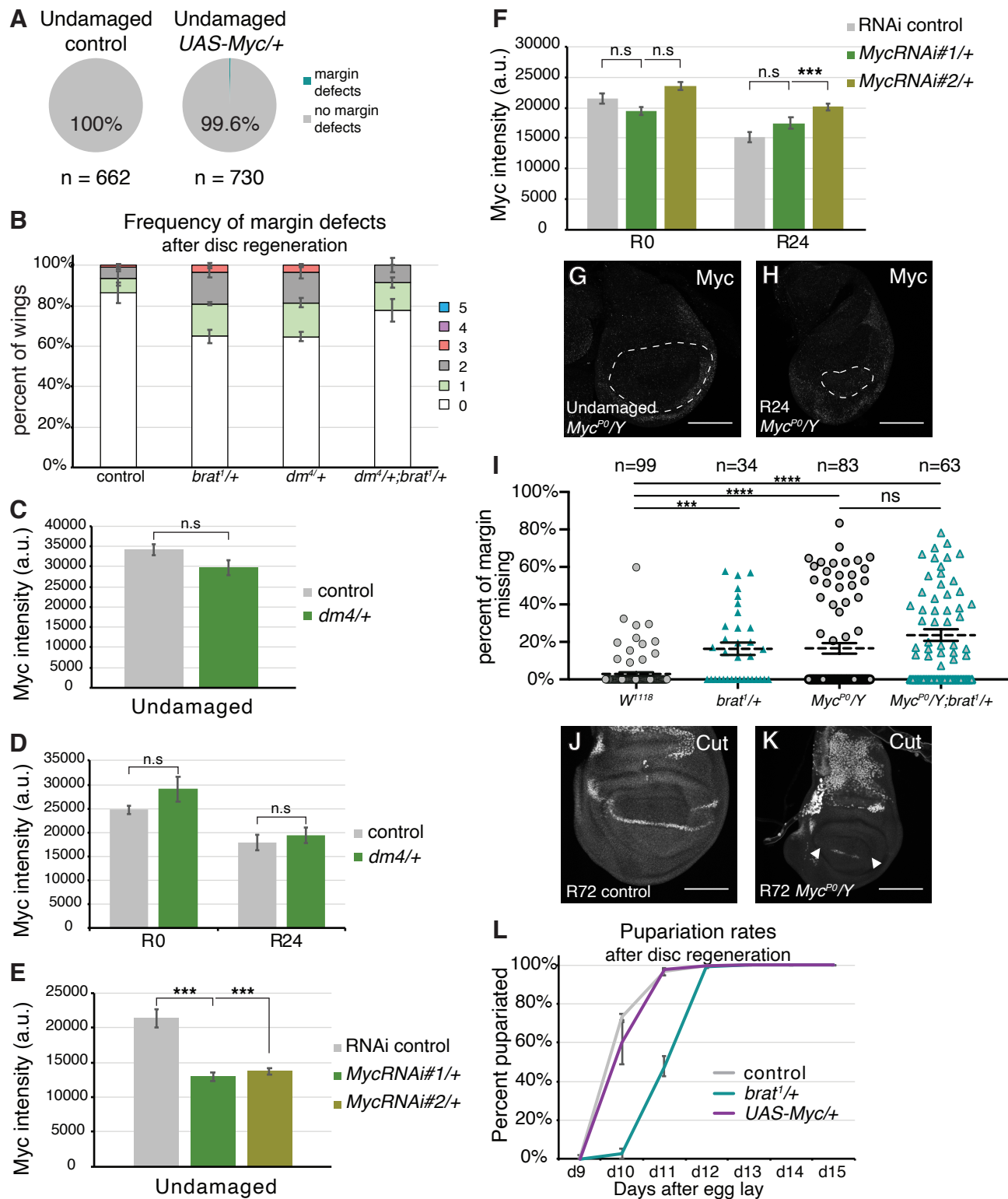

Supplement: S5 Fig — (A) Margin defects detected in adult wings from undamaged control (w1118) and UAS-Myc/+ discs. rnGAL4, GAL80ts/TM6B females were crossed to w1118 or UAS-Myc males and taken through the protocol shown in Fig 1A. (B) Frequency of margin defects in adult wings after disc regeneration for control (w1118) (n = 103), brat1/+ (n = 203), dm4/+ (n = 94) and dm4/+; brat1/+ (n = 94) wings, from three independent experiments. (C) Quantification of Myc fluorescence intensity in undamaged control (w1118) (n = 12) and dm4/+ (n = 11) discs. w1118 males were crossed to w1118 or dm4/FM7i, ActGFP females and dissected when the animals were third instar. Area for fluorescence intensity measurement was defined by wing pouch morphology and the elevated Myc expression domain in the wing pouch. (D) Quantification of Myc fluorescence intensity in R0 control (w1118) (n = 13), R0 dm4/+ (n = 10), R24 control (w1118) (n = 13), and R24 dm4/+ (n = 10) discs. Area for fluorescence intensity measurement was defined by the elevated Myc expression domain in the wing pouch. (E) Quantification of Myc fluorescence intensity in undamaged control (VDRC genetic background line, called control) (n = 14), MycRNAi#1/+ (n = 12), and MycRNAi#2/+ (n = 13) discs. rnGAL4, GAL80ts/TM6B females were crossed to the control, MycRNAi#1, or MycRNAi#2 males. The animals were shifted to 30°C during early third instar and kept there for 28 hours then dissected. MycRNAi#1/+ *** p < 0.000007, MycRNAi#2/+ *** p < 0.00002. Area for fluorescence intensity measurement was defined by wing pouch morphology. (F) Quantification of Myc fluorescence intensity in R0 control (n = 13), R0 MycRNAi#1/+ (n = 15), R0 MycRNAi#2/+ (n = 13), R24 control (n = 13), R24 MycRNAi#1/+ (n = 13), and R24 MycRNAi#2/+ (n = 13) discs. Fluorescence intensity was measured in the area marked by Anti-Nubbin immunostaining. *** p < 0.00007. (G,H) Anti-Myc immunostaining in undamaged (G) and regenerating R24 (H) MycP0/Y imaginal wing discs. Dashed line outlines [file pgen.1011103.s005.pdf]

**Figure S6**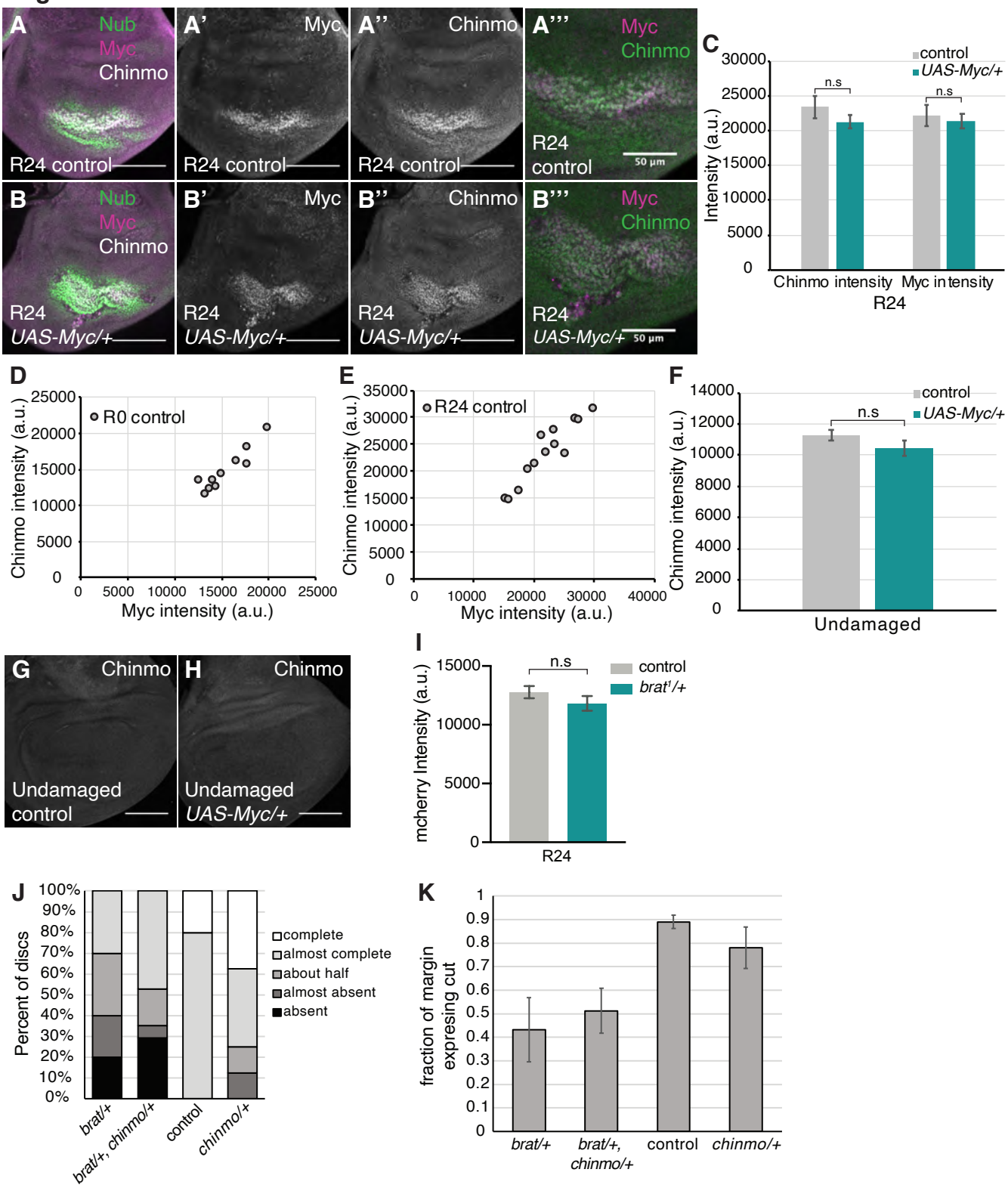

Supplement: S6 Fig — (A) Merge of anti-Nubbin, anti-Myc and anti-Chinmo immunostaining in an R24 control (w1118) disc. (A’-A”) Same disc as (A) showing anti-Myc and anti-Chinmo immunostaining, respectively. (A”’) Same disc as (A) showing an enlarged merge of anti-Myc and anti-Chinmo immunostaining. (B) Merge of anti-Nubbin, anti-Myc and anti-Chinmo immunostaining in an R24 UAS-Myc/+ disc. (B’-B”) Same disc as (B) showing anti-Myc and anti-Chinmo immunostaining, respectively. (B”’) Same disc as (B) showing an enlarged merge of anti-Myc and anti-Chinmo immunostaining. (C) Quantification of Chinmo and Myc fluorescence intensity in R24 control (w1118) (n = 13) and R24 UAS-Myc/+ (n = 14) discs. Area for fluorescence intensity measurement was defined by the elevated Myc expression domain in the wing pouch. Note that Myc and Chinmo expression co-localize. (D-E) Scatter plot showing correlation between Myc and Chinmo expression levels at R0 (D) and R24 (E). Pearson correlation coefficient for R0 = 0.93 and R24 = 0.94. (F) Quantification of Chinmo fluorescence intensity in undamaged discs dissected 31 hours after animals were shifted to 30°C on day 7 AEL. Control (+; rnGAL4,GAL80ts/+) (n = 14) and UAS-Myc (UAS-Myc/+; rnGAL4, GAL80ts/+) (n = 14). Area for fluorescence intensity measurement was defined by the Myc expression domain in the wing pouch. (G-H) Anti-Chinmo immunostaining in an undamaged control disc (G) and an undamaged UAS-Myc/+ disc (H). (I) Quantification of fluorescence intensity from the UAS-mCherry-chinmoUTR transgene in control (n = 16) and brat/+ (n = 15) R24 regenerating discs. Area for fluorescence intensity measurement was defined by Nubbin expression. (J) Quantification of wing discs with Ct expression patterns at R72 categorized as complete, almost complete, about half, almost absent, or absent in brat1/+ (n = 10), brat1/+, chinmo1/+ (n = 17), control w1118 (n = 10), and chinmo1/+ (n = 8) regenerating discs. (K) Quantification of the fraction of each disc margin that expres [file pgen.1011103.s006.pdf]
